# How to Promote the Performance of Parametric Volatility Forecasts in the Stock Market? A Neural Networks Approach

**DOI:** 10.3390/e23091151

**Published:** 2021-09-01

**Authors:** Jung-Bin Su

**Affiliations:** School of Finance, Qilu University of Technology, No. 3501, Daxue Road, Changqing District, Jinan 250353, China; jungbinsu@qlu.edu.cn; Tel.: +86-136-5862-8176

**Keywords:** volatility forecasting, neural networks, GARCH, skewed generalized Student’s t, stock market

## Abstract

This study uses the fourteen stock indices as the sample and then utilizes eight parametric volatility forecasting models and eight composed volatility forecasting models to explore whether the neural network approach and the settings of leverage effect and non-normal return distribution can promote the performance of volatility forecasting, and which one of the sixteen models possesses the best volatility forecasting performance. The eight parametric volatility forecasts models are composed of the generalized autoregressive conditional heteroskedasticity (GARCH) or GJR-GARCH volatility specification combining with the normal, Student’s t, skewed Student’s t, and generalized skewed Student’s t distributions. Empirical results show that, the performance for the composed volatility forecasting approach is significantly superior to that for the parametric volatility forecasting approach. Furthermore, the GJR-GARCH volatility specification has better performance than the GARCH one. In addition, the non-normal distribution does not have better forecasting performance than the normal distribution. In addition, the GJR-GARCH model combined with both the normal distribution and a neural network approach has the best performance of volatility forecasting among sixteen models. Thus, a neural network approach significantly promotes the performance of volatility forecasting. On the other hand, the setting of leverage effect can encourage the performance of volatility forecasting whereas the setting of non-normal distribution cannot.

## 1. Introduction

Volatility is a statistical measure of the dispersion of returns for a given asset. A higher volatility means that an asset’s price can change dramatically over a short time period in either direction, and thus is expected to be less predictable. On the other hand, a lower volatility means that an asset’s price does not fluctuate dramatically, and then tends to be more steady (volatility is often measured by either the standard deviation or variance between returns from that same asset). Hence, volatility can be used to measure the amount of uncertainty or risk related to the size of changes in an asset’s price, and it obeys the criteria: ‘the higher the volatility and then the riskier the asset’. Because of the above property for volatility, volatility is usually used in asset allocation [1,2,3], option pricing [4,5], risk management [6,7,8,9] and hedge strategy [10,11]. Thus, how to accurately predict the volatility of an asset is a very important issue in the actual investment process in the financial field. As to the issue of volatility forecasting, most of literatures used the generalized autoregressive conditional heteroskedasticity (GARCH) family models, a parametric volatility forecasting approach, to predict the volatility of an asset [12,13,14,15,16,17,18,19]. Because this type of model can capture most common features of financial assets such as both the linear dependence and strong autoregressive conditional heteroskedasticity (ARCH) effect subsisting on the return series, and both the volatility clustering and leverage effect usually existing at the volatility of financial asset returns series [8,20,21,22,23] (Volatility clustering means that large changes tend to be followed by large changes, of either sign, and small changes tend to be followed by small changes [23]. Conversely, the leverage effect is the extra increase of volatility caused by the bad news [20]. Notably, the volatility clustering and leverage effect appear significantly for financial assets, especially for the stock market [8,22]).

Regarding past literatures about volatility forecasting, they tried to use a more flexible model or a complex approach to increase the performance of volatility forecasting [15,16,17,19]. For example, Aliyev et al. [12] used the GARCH, exponential GARCH (EGARCH) and the GJR-GARCH model of Glosten, Jagannathan and Runkle [24] models with normal distribution (i.e., the GARCH-N, EGARCH-N and GJR-GARCH-N) to estimate the volatility of the Nasdaq-100. Chun et al. [14] used the above three models, the GARCH-N, EGARCH-N and GJR-GARCH-N, to forecast the volatility in the KOSPI200 in the Korean market. They found that the GJR-GARCH model has the best performance of volatility forecasting among the above three GARCH-family models. Lee and Pai [15] used the GARCH with the normal, skewed Student’s t and skewed generalized error distributions (SGED) (i.e., the GARCH-N, GARCH-ST and GARCH-SGED) to forecast the volatility of REIT in the United States. They found that the GARCH-SGED model is superior to the GARCH-N and GARCH-ST models. Liu and Hung [16] applied the GARCH-N, GARCH-T, GARCH-HT, GARCH-SGT, GJR-GARCH-N and EGARCH-N models to forecast the volatility of the Standard & Poor’s 100 stock index. They found that the GJR-GARCH-N model achieves the most accurate volatility forecasts, followed by the EGARCH-N model (the GARCH-T, GARCH-HT and GARCH-SGT models are the GARCH model with the Student’s t, heavy-tailed and skewed generalized Student’s t distributions, respectively). Lv and Shan [17] used the RiskMetrics, GARCH, IGARCH, GJR-GARCH, EGARCH, FIGARCH and FIEGARCH with the normal and skewed Student’s t distributions to forecast the spot and futures price volatilities of natural gas. They found that the simple linear GARCH-class models overwhelmingly outperform nonlinear models in forecasting spot price volatility. However, nonlinear models are also superior to linear models in forecasting future price volatility (the simple linear GARCH-class models include the RiskMetrics, GARCH and IGARCH models whereas the nonlinear models contain the GJR-GARCH, EGARCH, FIGARCH and FIEGARCH models. Moreover, the IGARCH is the integrated GARCH model whereas the FIGARCH is the fractionally integrated GARCH model. In addition, the FIEGARCH is the fractionally integrated EGARCH model). Su [18] applied the GARCH and GJR-GARCH model with the SGED distribution (GJR-GARCH-SGED) to forecast the volatility of six stock indices such as the NYSE, Brussels, CAC40, DAX, SWISS and NIKKEI. He found that asymmetric volatility specification (GJR-GARCH model) forecasts out-of-sample volatility more accurately than symmetric volatility specification (GARCH model). From the above discussion, I discovered the following phenomena: First, most of literatures only used one sample asset such as the Nasdaq-100 [12], KOSPI200 [14], REIT [15], Standard & Poor’s 100 [16] and natural gas [17], indicating that the results may not be credible because the number of sample assets is not enough. Second, the empirical model may be a model with a flexible volatility specification [12,14], generalized return distribution [15] or with both flexible volatility specification and generalized return distribution [16,17,18]. Moreover, among the flexible volatility specifications the GJR–GARCH model has the best performance of volatility forecasting [13,14,16,18]. In addition, the GJR-GARCH-SGED model in Su [18] is the most flexible model among the models with both flexible volatility specification and generalized return distribution [16,17,18]. Third, the volatility forecasting models in the above literatures all focus on the GARCH-family with alternative distributions—a parametric approach.

**Figure 1:**
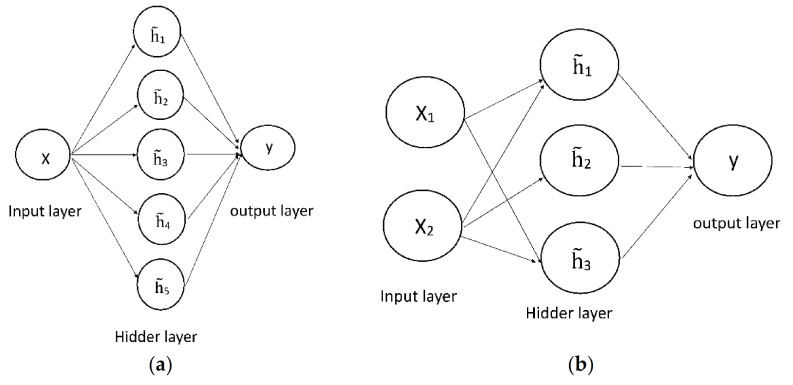
The structure of backpropagation neural network. (**a**) One backpropagation neural networks with an input layer including two input nodes ($x_{1}{\;\mathrm{and}\;x}_{2}$), a hidden layer of three hidden nodes (${\tilde{h}}_{1},{\tilde{h}}_{2}\;\mathrm{and}\;{\tilde{h}}_{3}$), and an output layer containing an output node (y). (**b**) One backpropagation neural networks with an input layer including one input node (x), a hidden layer of five hidden nodes (${\tilde{h}}_{1},{\tilde{h}}_{2},{\tilde{h}}_{3},{\tilde{h}}_{4},\mathrm{and}\;{\tilde{h}}_{5}$), and an output layer containing one output node (y).

Hence, in order to fill the gap of the above literatures, this study uses the fourteen stock indices as the sample assets and then utilizes a more flexible parametric volatility forecasting model combining with a neural network approach, an artificial intelligence technique, to promote the performance of volatility forecasting in the stock markets. The fourteen stock indices are the stock indices in the developed and emerging markets. This is the first contribution of this study because the number of sample assets is sufficient and the fourteen stock indices are distributed between countries with different degrees of economic development, indicating that the results obtained from this study are credible and representative in the stock market. The parametric volatility forecasting models in this study are mainly the asymmetric type of GARCH model, the GJR-GARCH of Glosten et al. [24], combined with the skewed generalized Student’s t (SGT) distribution of Theodossiou [25]. This is the second contribution of this study because the GJR-GARCH model with the SGT distribution (hereafter, the GJR-GARCH-SGT) is more flexible than the GJR-GARCH-SGED model in Su [18] and it can seize most of common features of financial assets such as, the volatility clustering and leverage effect existing on the volatility, and the skewness and kurtosis appearing on the return distribution (the GJR-GARCH model of Glosten et al. [24] can significantly seize the volatility clustering and leverage effect appearing at financial assets especially for the stock market. On the contrary, the skewed generalized Student’s t (SGT) distribution of Theodossiou [25] is able to capture the features of non-normal distribution such as the distribution of returns being left-skewed and having a larger and thicker tail than the normal distribution. In addition, the standardized SGT distribution degenerates into the standardized SGED distribution if n→∞). A neural network (NN) model is a multilayer perceptron, with one input layer, at least one hidden layer and one output layer, and one or more nodes within each layer. Moreover, each input node is connected to each hidden node, and in turn, each hidden node is connected to each output node (an artificial neural network is an interconnected group of nodes, inspired by a simplification of neurons in the brain. Here, each circular node represents an artificial neuron and an arrow represents a connection from the output of one artificial neuron to the input of another. Please see Figure 1 for more details). In other words, a neural network model is composed of an interconnected group of nodes. Hence, the structure of neural network models is similar to that of the network topology, and thus they both have the same concept (network topology is the topological structure of a network and may be depicted physically or logically. Physical topology is the placement of the various components of a network including several device locations and the cable installation between them. The device locations are regarded as the nodes of a network and the cable installation connects the device locations. Thus, the cable installation can be considered as the links or lines between the nodes in that network. On the other hand, logical topology illustrates how data flows within a network). Notably, because of the special training process of neural networks, a neural network approach is particularly useful for handling complex, non-linear univariate and multivariate relationships that would be difficult to fit using other techniques (in the training process of neural networks, these rules are initially determined by a set of initial weight values, and their weights are adjusted during the learning process to increase efficiency. Through continuous adjustment and learning, the true network output and target value are achieved. After reaching the same value, the weighted value in the network is fixed, and the training is considered complete). Hence, this study utilizes a neural network approach to promote the performance of volatility forecasts for alternative GARCH family models in the stock market. This is the third contribution of this study because the above literatures of volatility forecasting never use this approach.

Thus, this study uses the stock indices in the developed and emerging markets as the sample and then utilizes eight parametric volatility forecasting models and eight composed volatility forecasting models to explore whether the neural network approach can promote the performance of volatility forecasts, whether the settings of the leverage effect and return distribution can encourage the performance of volatility forecasts and which one of the 16 models possesses the best volatility forecasting performance. In addition, for each of fourteen stock indices, this study also investigates which model is the most suitable for it. The stock indices in the Group of Seven (G7) and Emerging Seven (E7) are used to represent the developed and emerging markets in this study, respectively (the Group of Seven (G7) is an intergovernmental organization consisting of the United States, Canada, the United Kingdom, France, Germany, Italy and Japan. Its members are the world’s largest IMF-advanced economies and wealthiest liberal democracies. On the other hand, the Emerging 7 (E7) is the seven countries Brazil, Russia, India, China, Mexico, Indonesia and Turkey. They have the highest economic performance in the class of emerging economies or they are the seven biggest emerging countries in terms of economic growth). The eight parametric volatility forecasting models are composed of the GJR-GARCH-SGT model and its seven degenerate models. The eight composed volatility forecasting models are obtained by eight parametric volatility forecasting models combined with a neural network approach. From the empirical results of both the preliminary analysis of data and the performance comparison of the 16 models, I discovered the following phenomena. The preliminary analysis of data includes the descriptive statistics of data and estimation results of the GJR-GARCH-SGT model. First, the stock indices in the E7 have higher return and higher risk than those in the G7. Second, all the stock indices in the G7 and E7 for the forecasting period have higher risk than those for the overall period because of COVID-19 spreading throughout the world in the last year. Third, the leverage effect is significant in the stock indices in the G7 and E7, especially for the G7. Fourth, the distribution of returns is left-skewed and has a larger and thicker tail than the normal distribution. Fifth, the performance for the composed volatility forecasting models is significantly superior to that for the parametric volatility forecasting models, and thus the neural network approach can significantly promote the performance of volatility forecasting. Sixth, the performance for the GJR-based models is significantly superior to that of the GARCH-based models, and thus the setting of the leverage effect can significantly encourage the performance of volatility forecasting. Seventh, the performance of the models with non-normal distribution is not superior to that of the models with normal distribution, and thus the setting of the non-normal return distribution cannot promote the performance of volatility forecasting. Eighth, among the 16 models in this study, the performance of the GJR-GARCH-N-NN models is the best followed by the GJR-GARCH-N, GJR-GARCH-T-NN and GJR-GARCH-SGT-NN, and thus the GJR-GARCH model combining both the normal distribution and a neural network approach has the better performance of volatility forecasting. Finally, for each of the 14 stock indices, the most suitable models are not necessarily the same but they possess the setting of leverage effect and further combine with a neural network approach, and thus these results are the same as those obtained from the analysis of previous issues.

The remainder of this paper is organized as follows. Section 2 describes the empirical models utilized in this study, eight parametric volatility forecasting models and eight composed volatility forecasting models, and two types of loss function to evaluate the above models. Section 3 states the basic statistical features of the return series for the stock indices in the E7 and G7 during the overall period and two sub-periods: estimation period and forecast period. Section 4 analyzes the results of the empirical model and further explores the issues addressed in this study. Finally, Section 5 concludes the findings in Section 3 and Section 4.

## 2. Methodology

In order to accurately forecast volatility, the selected empirical model should capture the common features of financial assets. For example, the distribution of returns is skewed to the right or left and has a larger and thicker tail than the normal distribution. In other words, the return series is not normally distributed. Moreover, the return series exhibits linear dependence and strong ARCH effect. In addition, the volatility clustering and leverage effect usually exists in the volatility of financial asset return series [8,20,23]. Hence, the empirical models include a symmetric type of volatility specification, the GARCH, and an asymmetric one, the GJR-GARCH, combined with the normal (N), Student’s t (T), skewed Student’s t (ST) and skewed generalized Student’s t (SGT) distributions, totaling eight different models (the GJR-GARCH model of Glosten et al. [24] is an asymmetric type of GARCH-based model, and it can seize the financial features: the volatility clustering and leverage effect. On the contrary, the asymmetric type of distributions, skewed Student’s t (ST) and skewed generalized Student’s t (SGT), can capture the skewness and fat-tails on the distribution of return). The eight models above can be divided into two categories. The first category of model includes the GARCH-N, GARCH-T, GARCH-ST and GARCH-SGT models, named the GARCH-based models. The second category of model consists of the GJR-GARCH-N, GJR-GARCH-T, GJR-GARCH-ST and GJR-GARCH-SGT models, called the GJR-based models. The eight models above are called parametric volatility forecasting models. Notably, this study combines the above parametric volatility forecasting models with a neural network (NN) approach to promote the volatility forecasting performance in the stock market. Hence, there are an additional eight models, the GARCH-N-NN, GARCH-T-NN, GARCH-ST-NN, GARCH-SGT-NN, GJR-GARCH-N-NN, GJR-GARCH-T-NN, GJR-GARCH-ST-NN and GJR-GARCH-SGT-NN models, respectively, representing the GARCH-N, GARCH-T, GARCH-ST, GARCH-SGT, GJR-GARCH-N, GJR-GARCH-T, GJR-GARCH-ST and GJR-GARCH-SGT models combined with a neural network approach. The above eight models are named as composed volatility forecasting models.

### 2.1. Parametric Volatility Forecasting Models

Among the eight parametric volatility forecasting models in this study, the GJR-GARCH-SGT model can degenerate into the other seven models. Hence, in this subsection, I mainly illustrate the mean and variance equations of the GJR-GARCH-SGT model and then describe how the GJR-GARCH-SGT model be degenerated into the other seven models. The mean and variance equations of the GJR-GARCH-SGT model are expressed as follows:

(1) $$r_{t}=\mu_{t}+e_{t},\;\mu_{t}=\phi_{0}+\phi_{1}r_{t-1},\;e_{t}=z_{t}\sqrt{h_{t}},\;z_{t}\sim \mathrm{IID}\;\mathrm{SGT}\left(0,1;\kappa ,\lambda ,n\right)$$

(2) $$h_{t}=\omega +\left(\alpha +{\eta I}_{t-1}^{-}\right)e_{t-1}^{2}+{\beta h}_{t-1}$$

where $r_{t}$ represents the return of the stock indices in the emerging and developed markets where $r_{t}=\left({\mathrm{lnP}}_{t}-{\mathrm{lnP}}_{t-1}\right)\times 100$. $P_{t}$ is the close price of the stock index at time t and $e_{t}$ is the current error. $\mu_{t}$ and $h_{t}$ represent the conditional mean and variance of return, respectively. Moreover, $I_{t-1}^{-}$ is an indicator dummy that takes the value of 1 if $e_{t-1}<0$ and zero otherwise, and thus parameter η is used to capture the leverage effect of volatility. Furthermore, ω, α and β are the parameters of variance equation and they obey the constraints ω, α, β>0 and β+α+0.5η<1 (according to Example 2.1 of Ling and McAleer [26], the necessary and sufficient condition for the existence of the second moment condition for all GJR-based models is β+α+0.5η<1). Notably, if η=0 in Equation (2), then the GJR-GARCH volatility specification degenerates into the GARCH volatility specification. IID denotes that the standardized errors $z_{t}$ are independent and identically distributed. Because $z_{t}$ is drawn from the standardized SGT distribution which allows returns innovation to follow a flexible treatment of both skewness and excess kurtosis in the conditional distribution of returns. The probability density function for the standardized SGT distribution is derived in Lee and Su [7] and can be represented as follows (the standardized SGT distribution, which has zero mean and unit variance, was checked by Mathematica software and another analogous standardized SGT distribution was proposed by Bali and Theodossiou [27]):

(3) $$f\left(z_{t}\right)=C{\left\{1+\frac{{\left|z_{t}+\delta \right|}^{\kappa }}{{\left[1+\mathrm{sign}\left(z_{t}+\delta \right)\lambda \right]}^{\kappa }\theta^{\kappa }}\right\}}^{-\frac{n+1}{\kappa }}$$

where $\theta =\frac{1}{S\left(\lambda \right)}B{\left(\frac{1}{\kappa },\frac{n}{\kappa }\right)}^{\frac{1}{2}}B{\left(\frac{3}{\kappa },\frac{n-2}{\kappa }\right)}^{-\frac{1}{2}}$, $S\left(\lambda \right)=\sqrt{1+3\lambda^{2}-4A^{2}\lambda^{2}}$, $A=B\left(\frac{2}{\kappa },\frac{n-1}{\kappa }\right)B{\left(\frac{1}{\kappa },\frac{n}{\kappa }\right)}^{-\frac{1}{2}}B{\left(\frac{3}{\kappa },\frac{n-2}{\kappa }\right)}^{-\frac{1}{2}}$, $\delta =\frac{2\lambda A}{S\left(\lambda \right)}$, $C=\frac{\kappa }{2\theta }B{\left(\frac{1}{\kappa },\frac{n}{\kappa }\right)}^{-1}$.

where κ, n and λ are the scaling parameters and C and θ are the normalizing constants ensuring that $f\left(\cdot \right)$ is a proper probability density function. The parameters κ and n control the height and tails of density with the constraints κ>0 and n>2, respectively. The skewness parameter λ controls the rate of descent of the density around the mode of $z_{t}$ with −1<λ<1. In the case of positive and negative skewness, the density function skews toward to the right and left, respectively. $B\left(\cdot \right)$ is the beta function whereas ‘sign’ denotes a sign function. The parameter n has the degrees of freedom interpretation in case λ=0 and κ=2. The log-likelihood function of the GJR-GARCH-SGT model thus can be expressed as:

(4) $$L\left(\Psi \right)=\ln f\left(r_{t}\left|\Omega_{t-1};\Psi \right.\right)\;=\ln C-\ln\sqrt{h_{t}}-\frac{n+1}{\kappa }\ln\left\{1+{\left|\frac{r_{t}-\mu_{t}}{\sqrt{h_{t}}}+\delta \right|}^{\kappa }{\left[1+\mathrm{sign}\left(\frac{r_{t}-\mu_{t}}{\sqrt{h_{t}}}+\delta \right)\lambda \right]}^{-\kappa }\theta^{-\kappa }\right\}$$

where $\Psi =\left[\phi_{0},\phi_{1},\;\omega ,\alpha ,\beta ,\eta ,\kappa ,\lambda ,n\right]$ is the vector of parameters to be estimated, and $\Omega_{t-1}$ denotes the information set of all observed returns up to time t−1. Notably, if κ=2 in Equation (3), then the standardized SGT distribution degenerates into the standardized ST distribution. Using the same inference process, the standardized SGT distribution degenerates into the standardized Student’s t distribution if κ=2 and λ=0 and it also degenerates into the standardized normal distribution if κ=2, λ=0 and n→∞ (regarding the process of the SGT distribution degenerating into the normal, Student’s t, skewed Student’s t (ST) distributions, please see Lee and Su [7] for more details).

### 2.2. Composed Volatility Forecasting Models

The eight composed volatility forecasting models are the GARCH-N-NN, GARCH-T-NN, GARCH-ST-NN, GARCH-SGT-NN, GJR-GARCH-N-NN, GJR-GARCH-T-NN, GJR-GARCH-ST-NN and GJR-GARCH-SGT-NN models. They are obtained by eight parametric volatility forecasting models combined with a neural network approach. A neural network (NN) approach is particularly useful for handling the complex, non-linear univariate and multivariate relationships that would be difficult to fit by using other techniques. A neural network model is composed of a multilayer perceptron with an interconnected group of nodes. For example, Figure 1a shows one backpropagation neural network with three layers, an input layer including two input nodes ($x_{1}{\;\mathrm{and}\;x}_{2}$), a hidden layer of three hidden nodes (${\tilde{h}}_{1},{\tilde{h}}_{2}\;\mathrm{and}\;{\tilde{h}}_{3}$) and an output layer containing an output node (y). On the other hand, Figure 1b displays the other backpropagation neural network with an input layer including one input node (x), a hidden layer of five hidden nodes (${\tilde{h}}_{1},{\tilde{h}}_{2},{\tilde{h}}_{3},{\tilde{h}}_{4},\mathrm{and}\;{\tilde{h}}_{5}$) and an output layer containing one output node (y). Notably, the input nodes and the output nodes are analogous to the explanatory variables and the dependent variables in a regression model, respectively. The theory of neural network (NN) models is illustrated as follows (regarding the theory of neural network (NN) models, please see Lu et al. [28] and chapter 12 in the user’s guide of RATS version 6 or Doan [29]): Subsequently, I used a vector to represent the nodes of each layer and the total number of elements in a vector denote that of node in a layer. For example, the vectors $X=\left(x_{1},x_{2},\ldots ,x_{d}\right)$ and $Y=\left(y_{1},y_{2},\ldots ,y_{c}\right)$ represent the nodes in the input layer and output layer, respectively. On the other hand, the vector **H**$=\left({\tilde{h}}_{1},{\tilde{h}}_{2},\ldots ,{\tilde{h}}_{m}\right)$ denotes the nodes in the hidden layer. The three layers’ perceptron model is obtained by a weighted linear combination of the d input values from the d input nodes, $X=\left(x_{1},x_{2},\ldots ,x_{d}\right)$, and is expressed as follows:

(5) $$a_{j}=\sum_{i=1}^{d}w_{\mathrm{ji}}^{\left(1\right)}x_{i}\cdot$$

The activation of hidden unit j can be achieved by transforming the linear sum via using a logistic activation function $g\left(a_{j}\right)=1/\left(1+e^{-a_{j}}\right)$:

(6) $$\tilde{h_{j}}=g\left(a_{j}\right)=g\left(\overset{d}{\underset{i=1}{\sum }}w_{\mathrm{ji}}^{\left(1\right)}x_{i}\right)$$

Thus, the node of output layer is defined as:

(7) $$y_{k}=\tilde{g}\left(\overset{m}{\underset{j=1}{\sum }}w_{\mathrm{jk}}^{\left(2\right)}g\left(\overset{d}{\underset{i=1}{\sum }}w_{\mathrm{ji}}^{\left(1\right)}x_{i}\right)\right)$$

**Figure 2:**
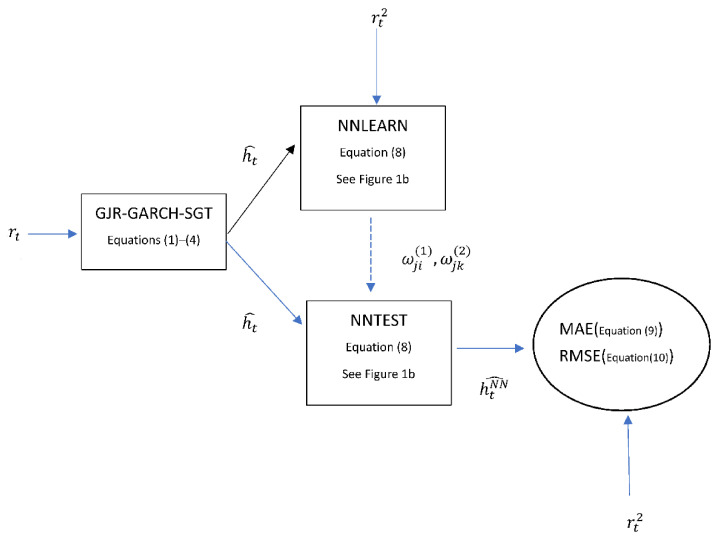
The schematic diagram for the volatility forecasting process of a neural network approach.

If the output function is taken linear, $\tilde{g}\left(a\right)=a$, the output model reduces to:

(8) $$y_{k}=\sum_{j=1}^{m}w_{\mathrm{jk}}^{\left(2\right)}g\left(\sum_{i=1}^{d}w_{\mathrm{ji}}^{\left(1\right)}x_{i}\right)\cdot$$

where $w_{\mathrm{ji}}^{\left(1\right)}$ and $w_{\mathrm{jk}}^{\left(2\right)}$ are the weights of the hidden node and output node, respectively. Notably, fitting a neural networks model involves a training process of this model. The training process is executed by supplying a set of known input and output values, and then allowing the neural networks algorithm to adjust the hidden node and output node weights until the output produced by the networks matches the actual output in the training sample to the desired degree of accuracy (in this study, the input values are a volatility forecasting series ${\hat{h}}_{t}$ obtained by the variance equation of parametric volatility forecasting models as shown in Equation (2). On the contrary, the output values are the true values of variance and are replaced by the squared intraday return series ($r_{t}^{2}$) as the proxies. In addition, the neural networks algorithm in the training process is the instruction of ‘NNLEARN’ in RATS version 6, manufacturer, city and country). Once the training process is completed, the model can be used to generate new output data from the other sets of inputs (the new output data may be the fitted values for the in-sample volatility forecasts or the forecast values for the out-of-sample volatility forecasts in this study. Notably, the new output data is obtained by the instruction of ‘NNTEST’ in RATS version 6). Assuming that the fit is good, the relationships represented by the sample input and output data can generalize to other samples, thus the model can produce good predictions. Thus, the volatility forecasts of the GJR-GARCH-SGT-NN model are obtained by assigning a volatility forecasting series ${\hat{h}}_{t}$ obtained from the GJR-GARCH-SGT model as the input values and using a backpropagation neural network with one input node, five hidden nodes and an output node as shown in Figure 1b. Thus, I took an example of the GJR-GARCH-SGT-NN model to explain the process of the volatility forecasting for a neural network approach. Figure 2 lists the schematic diagram for the process of the volatility forecasting of the GJR-GARCH-SGT-NN model, and is illustrated as follows:

Step 1  Fit a return series ($r_{t}$) into the GJR-GARCH-SGT model (see, Equations (1)–(4)). Then, get a forecasted variance series (${\hat{h}}_{t}$) (see, Equation (2))
Step 2  Input a forecasted variance series (${\hat{h}}_{t}$) obtained from step 1 and the true variance series ($r_{t}^{2}$) into the ‘NNLEARN’ function. Then, obtain the weights of the hidden nodes ($w_{\mathrm{ji}}^{\left(1\right)}$) and the weights of the output node ($w_{\mathrm{jk}}^{\left(2\right)}$). The ‘NNLEARN’ function respectively regards ${\hat{h}}_{t}$ and $r_{t}^{2}$ as the input value and output value of Figure 1b, and then substitutes them (${\hat{h}}_{t}$ and $r_{t}^{2}$) into Equation (8) to execute a training process of the neural network in order to obtain the weights $w_{\mathrm{ji}}^{\left(1\right)}$ and $w_{\mathrm{jk}}^{\left(2\right)}$ (in the training process of neural networks, the weights of the hidden nodes and the output node ($w_{\mathrm{ji}}^{\left(1\right)}$ and $w_{\mathrm{jk}}^{\left(2\right)}$) are initially determined by a set of initial weights values, and their weights are adjusted until the output produced by the networks (${\hat{h}}_{t}$) matches the actual output ($r_{t}^{2}$) in the training sample to the desired degree of accuracy. Te training is then considered complete, the weights $w_{\mathrm{ji}}^{\left(1\right)}$ and $w_{\mathrm{jk}}^{\left(2\right)}$ are obtained). Figure 1b is a structure of a backpropagation neural network with one node in the input layer, five nodes in the hidden layer and one node in the output layer.
Step 3  Input a forecasted variance series (${\hat{h}}_{t}$) obtained from step 1 and the weights of the hidden nodes ($w_{\mathrm{ji}}^{\left(1\right)}$) and the weights of the output node ($w_{\mathrm{jk}}^{\left(2\right)}$) obtained from step 2 into the ‘NNTEST’ function. Furthermore, obtain the forecasted variance series for a neural network approach (${\hat{h}}_{t}^{\mathrm{NN}}$). That is, the ‘NNTEST’ function substitutes the input value ${\hat{h}}_{t}$ and weights of the hidden nodes and output node ($w_{\mathrm{ji}}^{\left(1\right)}$ and $w_{\mathrm{jk}}^{\left(2\right)}$) into Equation (8), and then the output value ${\hat{h}}_{t}^{\mathrm{NN}}$ is obtained.
Step 4  Given a forecasted variance series for a neural network approach (${\hat{h}}_{t}^{\mathrm{NN}}$) and the true variance series ($r_{t}^{2}$), then calculate the values of loss functions, MAE and RMSE (see Equations (9) and (10)).

In addition, I used two types of loss function to perform the performance comparison of volatility forecasting for the sixteen models or four categories of model. The first category of model is composed of one parametric volatility forecasting model and one corresponding composed volatility forecasting model (for example, the GARCH-N and GARCH-N-NN, the GARCH-T and GARCH-T-NN, the GARCH-ST and GARCH-ST-NN, the GARCH-SGT and GARCH-SGT-NN, the GJR-GARCH-N and GJR-GARCH-N-NN, the GJR-GARCH-T and GJR-GARCH-T-NN, the GJR-GARCH-ST and GJR-GARCH-ST-NN and the GJR-GARCH-SGT and GJR-GARCH-SGT-NN, totaling 8 paired models in the first category of model). The second category of model is composed of one GARCH-based model and one corresponding GJR-based model (for instance, the GARCH-N and GJR-GARCH-N, the GARCH-T and GJR-GARCH-T, the GARCH-ST and GJR-GARCH-ST, the GARCH-SGT and GJR-GARCH-SGT, the GARCH-N-NN and GJR-GARCH-N-NN, the GARCH-T-NN and GJR-GARCH-T-NN, the GARCH-ST-NN and GJR-GARCH-ST-NN and the GARCH-SGT-NN and GJR-GARCH-SGT-NN, totaling 8 paired models in the second category of model). The third category of model is a group of models with the same volatility specification and volatility forecasting approach but different distribution (for example, the GARCH-N, GARCH-T, GARCH-ST and GARCH-SGT; the GJR-GARCH-N, GJR-GARCH-T, GJR-GARCH-ST and GJR-GARCH-SGT; the GARCH-N-NN, GARCH-T-NN, GARCH-ST-NN and GARCH-SGT-NN; and the GJR-GARCH-N-NN, GJR-GARCH-T-NN, GJR-GARCH-ST-NN and GJR-GARCH-SGT-NN, totaling four groups of model in the third category of model). The fourth category of model is composed of all sixteen models in this study. Hence, the performance comparison of volatility forecasting for the first category of model can explore whether the neural network approach can promote the performance of volatility forecasting. On the contrary, the performance comparison of volatility forecasting for the second and third categories of model can investigate whether the settings of the leverage effect and return distribution can encourage the performance of volatility forecasts, respectively. In addition, the performance comparison of volatility forecasting for the fourth category of model can explore which one of the 16 models possesses the best volatility forecasting performance. Two types of loss function are the mean absolute error (MAE) and root mean squared error (RMSE). The MAE measures the average magnitude of the errors in a set of forecasts, without considering their direction. The MAE for the out-of-sample volatility forecasting can be evaluated by the following equation.

(9) $$\mathrm{MAE}=\frac{1}{T^{\prime }}\sum_{i=1}^{T^{\prime }}\left|\varepsilon_{i}\right|=\frac{1}{T^{\prime }}\sum_{i=1}^{T^{\prime }}\left|{\hat{h}}_{t+i\left|t+i-1\right.}-h_{t+i}\right|$$

where $\varepsilon_{i}$ denotes the forecast error; ${\hat{h}}_{t+i\left|t+i-1\right.}$ is the one-step-ahead forecast of the variance of the returns dependent on all information upon the time t+i-1 and can be estimated by one of the sixteen models in this study; $h_{t+i}$ is the true value of variance and is replaced by the squared intraday returns as the proxies; $T^{\prime }$ is the number of computing 1-day-ahead variance and is equal to 250 in this study. Moreover, the RMSE for the out-of-sample volatility forecasting can be obtained by the following equation (the MAE and RMSE for in-sample volatility forecasting can be evaluated by the equation: $\mathrm{MAE}=\frac{1}{T^{\prime }}\sum_{i=1}^{T^{\prime }}\left|\varepsilon_{i}\right|$ = $\frac{1}{T^{\prime }}\sum_{i=1}^{T^{\prime }}\left|{\hat{h}}_{i}-h_{i}\right|$ and $\mathrm{RMSE}=\sqrt{\frac{1}{T^{\prime }}\sum_{i=1}^{T^{\prime }}{\left(\varepsilon_{i}\right)}^{2}}=\sqrt{\frac{1}{T^{\prime }}\sum_{i=1}^{T^{\prime }}{\left({\hat{h}}_{i}-h_{i}\right)}^{2}}$ where $T^{\prime }$ is the total number of observations for overall period and is equal to 3501 in this study).

(10) $$\mathrm{RMSE}=\sqrt{\frac{1}{T^{\prime }}\sum_{i=1}^{T^{\prime }}{\left(\varepsilon_{i}\right)}^{2}}=\sqrt{\frac{1}{T^{\prime }}\sum_{i=1}^{T^{\prime }}{\left({\hat{h}}_{t+i\left|t+i-1\right.}-h_{t+i}\right)}^{2}}$$

where $\varepsilon_{i}$, ${\hat{h}}_{t+i\left|t+i-1\right.}$, $h_{t+i}$ and $T^{\prime }$ are defined the same as for Equation (9). Hence, the MAE and RMSE can measure the differences between the values predicted by a model and the values actually observed from the thing being modeled or estimated.

## 3. Data and Descriptive Statistics

This study uses the stock indices in the developed and emerging markets as the sample to explore the issue of ‘how to promote the performance of parametric volatility forecasting models? A neural networks approach’. I used the stock indices in the G7 and E7 to represent the stock markets in the developed and emerging markets, respectively. The stock indices in the G7 include the Dow Jones (DJ), TSX, FTSE, CAC40, DAX, MIB and N225, respectively corresponding to the United States, Canada, the United Kingdom, France, Germany, Italy and Japan. On the contrary, the stock indices in the E7 are the BVSP, RTSI, BSE, SSE, MXX, JKSE and XU100, respectively corresponding to Brazil, Russia, India, China, Mexico, Indonesia and Turkey.

**Figure 3:**
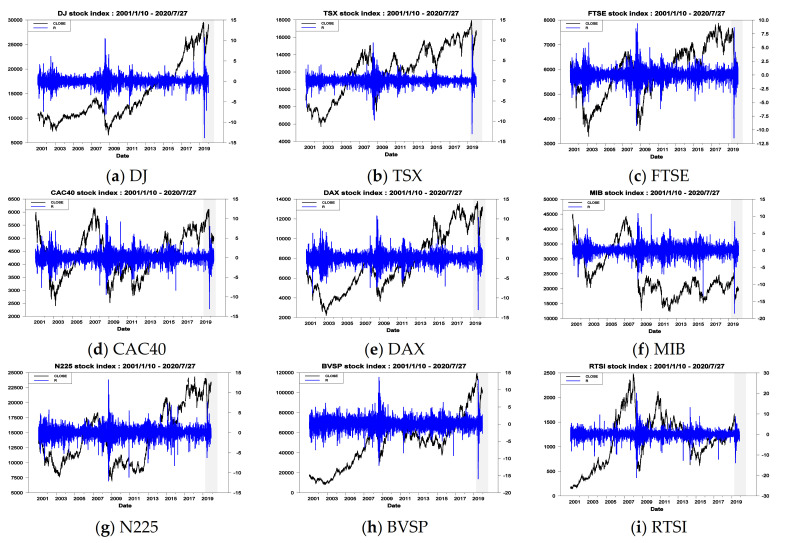

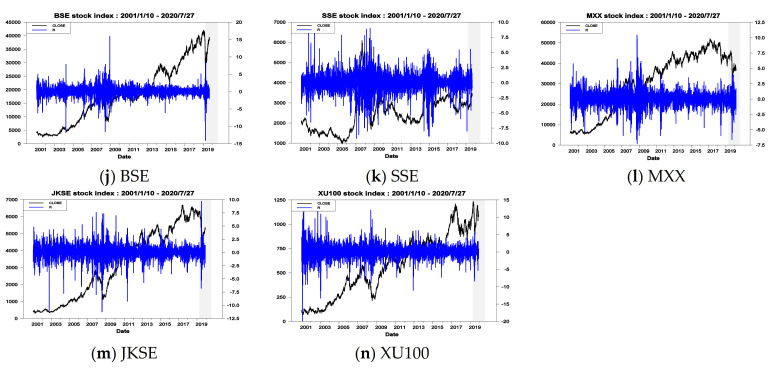
The price levels and returns of spot prices for overall period.

Table 1 reports basic descriptive statistics of daily return of stock indices in the G7 and E7 during the overall period and two sub-periods. The overall period starts from 10 January 2001 and ends 27 July 2020 and is used to perform the in-sample volatility forecasting. The overall period is divided into two sub-periods, the estimation period and forecasting period, to execute the out-of-sample volatility forecasting. The estimation period is from 10 January 2001 to 8 April 2019 whereas the forecasting period is from 9 April 2019 to 27 July 2020. Notably, all the daily close price data of the 14 stock indices were obtained from the Yahoo finance website. As shown in panel A of Table 1, the mean values of G7 are between −0.0215 (MIB) and 0.0263 (DJ) whereas those of E7 range from 0.0254 (SSE) to 0.0717 (JKSE). Conversely, the values of standard deviation for the G7 are between 1.2631 (TSX) and 1.8306 (MIB) whereas those for the E7 range from 1.4361 (MXX) to 2.3155 (RTSI). These results indicate that during the overall period, the stock indices in the E7 have the higher return and higher risk than those in the G7 (the reason is that the maximum value of mean for the E7 (0.0717) is greater than that for the G7 (0.0263) and the minimum value of mean for the E7 (0.0254) is also greater than that for the G7 (−0.0215). On the other hand, the maximum value of standard deviation for the E7 (2.3155) is greater than that for the G7 (1.8306) and the minimum value of standard deviation for the E7 (1.4361) is also greater than that for the G7 (1.2631)). The above finding is consistent with that found in Su [9]. As illustrated in panel B and panel C of Table 1, I discovered that the stock indices in the G7 and E7 for the estimation and forecasting periods possess the same phenomena found from the overall period. That is, the stock indices in the E7 have higher return and higher risk than those in the G7. Notably, regarding the forecasting period, the values of standard deviation for the G7 are between 1.7269 (N225) and 2.3058 (MIB) whereas those for the E7 range from 1.6095 (MXX) to 3.1898 (SSE). These results indicate that the stock indices in the G7 and E7 for the forecasting period have higher risk than those for the overall period (the reason is, regarding the G7, the maximum value of standard deviation for the forecasting period (2.3058) is greater than that for the overall period (1.8306) and the minimum value of standard deviation for the forecasting period (1.7269) is greater than that for the overall period (1.2631). Conversely, regarding the E7, the maximum value of standard deviation for the forecasting period (3.1898) is greater than that for the overall period (2.3155) and the minimum value of standard deviation for the forecasting period (1.6095) is greater than that for the overall period (1.4361)). This phenomenon is attributed to COVID-19 spreading throughout the world in the last year. Figure 3 illustrates the trend of price levels, and the variation of return for the 14 stock indices during the overall period. From Figure 3, I also discovered that in the last year, the price of stock indices underwent a severe decline and its return experienced a serious variation, indicating that the high risk appears at the forecasting period. This phenomenon is the same as that found from the above analysis. In addition, the volatility clustering occurs significantly in the overall period.

Finally, regarding the other descriptive statistics, they have the same features as those for most of the financial return series. For example, the distribution of returns is left-skewed and has a larger and thicker tail than the normal distribution, indicating that the return series is not normally distributed. The above results are found by coefficient of skewness, excess kurtosis and the J-B normality test statistics [30]. In addition, the return series exhibit linear dependence and strong ARCH effect as shown by the Ljung–Box $Q^{2}\left(20\right)$ statistics for the squared returns. From the above findings, a GARCH family model is very suitable to seize the fat tails and time-varying volatility found in these asset return series.

## 4. Empirical Results

As described in Section 2, the empirical models in this study can be divided into two categories: the parametric volatility forecasting models and composed volatility forecasting models. The composed volatility forecasting models are the parametric volatility forecasting models combined with a neural network approach. The parametric volatility forecasting models include the GARCH-N, GARCH-T, GARCH-ST, GARCH-SGT, GJR-GARCH-N, GJR-GARCH-T, GJR-GARCH-ST and GJR-GARCH-SGT models. Among the above eight parametric volatility forecasting models, the GJR-GARCH-SGT model is the most flexible because this model can capture most of the common features of financial assets and this model can degenerate into the other seven models under the setting of some restrictions. Hence, I will report some financial features for the 14 stock indices via using the empirical results of the GJR-GARCH-SGT model.

### 4.1. Estimation Results for the GJR-GARCH-SGT Model

Table 2 illustrates the empirical results of the GJR-GARCH-SGT model for the stock indices in the G7 and E7. As shown in Table 2, parameters ω, α and β are significantly positive for most stock indices and all stock indices obey the constraint β+α+0.5η<1 as reported by the numbers listed in row ‘C’ of Table 2. For example, the constraint β+α+0.5η for BVSP is equal to 0.9756, and is less than 1 because the values of parameters β, α and η are equal to 0.9197, 0.0143 and 0.0832, respectively. Moreover, parameter η is significantly positive at 1% for all stock indices. The values of parameter η for the stock indices in the G7 are greater than 0.1421 (TSX) whereas those for the stock indices in the E7 are smaller than 0.0873 (MXX) except for BSE. These results indicate that the leverage effect exists significantly in the stock indices in the G7 and E7, especially for the G7 because the values of parameter η for the case of G7 are greater than those for the E7. In addition, the shape parameters κ, n and λ are significant for most of stock indices and obey the constraints κ>0, n>2 and −1<λ<1. Notably, the values of parameter λ are significantly negative for most of stock indices. These results indicate that the distribution of returns is left-skewed and has a larger and thicker tail than the normal distribution. Finally, regarding the Ljung–Box *Q*^2^ (20) statistics for the squared returns, they are not significant at the 10% level for most of cases and they are all far smaller than the same statistics appearing in Table 1. These results indicate that the serial correlation does not exist in standard residuals and the GJR-GARCH-SGT model is sufficient to correct the serial correlation of these returns series in the conditional variance equation for the stock indices in the G7 and E7.

### 4.2. The Performance Assessment of Volatility Forecasts

**Figure 4:**
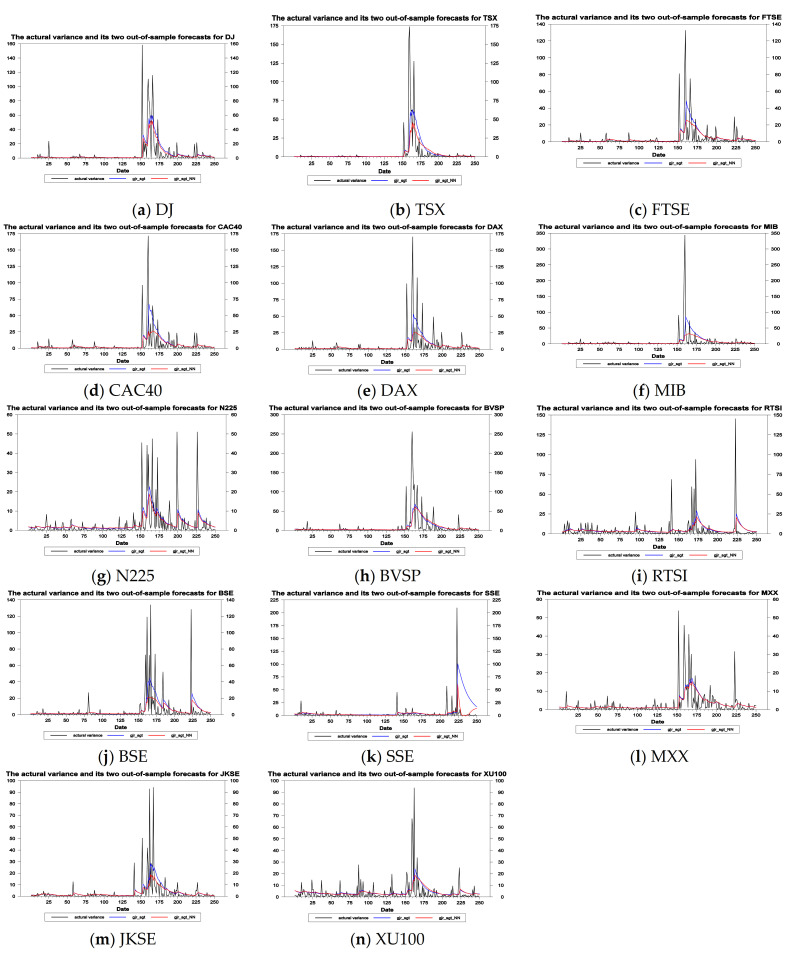
The actual variance and its two out-of-sample forecasts (the GJR-GARCH-SGT and GJR-GARCH-SGT-NN models).

Via the analysis of empirical results in Table 2, the selected empirical model in this study can capture the common features of financial assets well. I then executed the in-sample and out-of-sample volatility forecasts of the 16 models for the the 14 stock indices in the G7 and E7. Table 3 and Table 4 report the results of in-sample volatility forecasts, respectively, based on the MAE and RMSE loss functions for the overall period. On the contrary, Table 5 and Table 6 list the results of out-of-sample volatility forecasts, respectively, based on the MAE and RMSE loss functions for the forecasting period via using a rolling window approach (for each data series, the eight parametric volatility forecasting models and eight composed volatility forecasting models, totaling sixteen models, were first estimated using a sample of 3250 daily returns, and a volatility forecast for the next period was obtained. Subsequently, the estimation period was rolled forward by adding one new day and omitting the most distant day. By repeating this procedure, the out-of-sample volatility forecasts were calculated for the next 250 days). Figure 4 shows the trend of actual variance and its two out-of-sample variance forecasts obtained by the GJR-GARCH-SGT and GJR-GARCH-SGT-NN models. From Figure 4, I observed that there is a significantly sharp value of variance on March of last year owing to COVID-19 spreading througout the world.

Subsequently, regarding Table 3, Table 4, Table 5 and Table 6, I performed the volatility forecasting performance comparison for four categories of model to explore whether the neural network approach can promote the performance of volatility forecasting, whether the settings of leverage effect and non-normal return distribution can encourage the performance of volatility forecasting and which one of the 16 models possesses the best volatility forecasting performance, and then record the results of performance comparison in columns S1, S2, S3 and S4 of each table (regarding four categories of model, please see Section 2.2 for more details). The results in columns S1, S2, S3 and S4 of Table 3, Table 4, Table 5 and Table 6 are also summarized in Table 7 in order to easily explore the four main issues of this study. I used the data in Table 3 to illustrate the performance comparison of volatility forecasting for the four categories of the model. Regarding the performance comparison of volatility forecasting for the first category of model, I took the following two examples in Table 3 to illustrate it. First, regarding the paired models, ‘the GARCH-N and GARCH-N-NN’, the GARCH-N model has the lower value of MAE for the cases of DJ, TSX, FTSE and BVSP whereas the GARCH-N-NN model possesses the lower value of MAE for the cases of the other ten stock indices. For example, regarding the DJ, the value of MAE for the GARCH-N model (1.94301) is lower than that for the GARCH-N-NN model (1.94312). Furthermore, in Table 3 the results ‘4’ for the GARCH-N model and ‘10’ for the GARCH-N-NN model are recorded in column ‘S1’, respectively corresponding to the rows ‘GARCH-N’ and ‘GARCH-N-NN’. In Table 7, the above results, ‘4’ and ‘10’, are also recorded in column ‘MAE’ below ‘In-sample’ of S1, respectively corresponding to the rows ‘GARCH-N’ and ‘GARCH-N-NN’. Second, regarding the paired models, ‘the GJR-GARCH-SGT and GJR-GARCH-SGT-NN’, the GJR-GARCH-SGT-NN model possesses the lower value of MAE for all 14 stock indices but the GJR-GARCH-SGT model does not obtain the lower value of MAE. In Table 3 the results ‘0’ and ‘14’ are then recorded in column ‘S1’, respectively corresponding to the rows ‘GJR-GARCH-SGT’ and ‘GJR-GARCH-SGT-NN’. In Table 7, the above results, ‘0’ and ‘14’, are also recorded in column ‘MAE’ below ‘In-sample’ of S1, respectively corresponding to the rows ‘GJR-GARCH-SGT’ and ‘GJR-GARCH-SGT-NN’. Regarding the performance comparison of volatility forecasting for the second category of model, I took the following example in Table 3 to explain it. Regarding the paired models, ‘the GARCH-N and GJR-GARCH-N models’, the GARCH-N model has the lower value of MAE for the cases of MIB, RTSI, BSE and XU100 whereas the GJR-GARCH-N model possesses the lower value of MAE for the other ten stock indices. For example, regarding the MIB, the value of MAE for the GARCH-N model (3.79126) is lower than that for the GJR-GARCH-N model (3.79163). In Table 3 the results ‘4’ and ‘10’ are then recorded in column ‘S2’, respectively corresponding to the rows ‘GARCH-N’ and ‘GJR-GARCH-N’. In Table 7, the above results, ‘4’ and ‘10’, are also recorded in column ‘MAE’ below ‘In-sample’ of S2, respectively corresponding to the rows ‘GARCH-N’ and ‘GJR-GARCH-N’. Regarding the performance comparison of volatility forecasting for the third category of model, I took the following example in Table 3 to explain it. Among a group of models, ‘the GARCH-N, GARCH-T, GARCH-ST and GARCH-SGT’, the GARCH-N model possesses the lowest value of MAE for the cases of DJ, TSX, FTSE, CAC40, DAX, BVSP and RTSI but the GARCH-T model never obtains the lowest value of MAE. For instance, regarding the DJ, the value of MAE for the GARCH-N model (1.94301) is lower than that for the other three GARCH-based models such as the GARCH-T (1.99808), GARCH-ST (1.98032) and GARCH-SGT (1.95943). In other words, regarding the DJ, the GARCH-N model possesses the lowest value of MAE among four GARCH-based models. In addition, the GARCH-ST model has the lowest value of MAE for the cases of BSE, MXX and XU100 whereas the GARCH-SGT model obtains the lowest value of MAE for the cases of MIB, N225, SSE and JKSE. In Table 3 the results ‘7’, ‘0’, ‘3’, and ‘4’ are then recorded in column ‘S3’, respectively corresponding to the rows ‘GARCH-N’, ‘GARCH-T’, ‘GARCH-ST’ and ‘GARCH-SGT’. In Table 7, the above results ‘7’, ‘0’, ‘3’, and ‘4’ are also recorded in column ‘MAE’ below ‘In-sample’ of S3, respectively corresponding to the rows ‘GARCH-N’, ‘GARCH-T’, ‘GARCH-ST’ and ‘GARCH-SGT’. Regarding the performance comparison of volatility forecasting for the fourth category of model, I took the following example in Table 3 to explain it. Among all 16 models, the GJR-GARCH-N model has the lowest value of MAE for the cases of TSX and BVSP. For example, regarding the TSX, the value of MAE for the GJR-GARCH-N model (1.64781) is lower than that for the other fifteen models such as the GARCH-N (1.67592), GARCH-T (1.72006), GARCH-ST (1.70677), GARCH-SGT (1.70920), GJR-GARCH-T (1.69383), GJR-GARCH-ST (1.68906), GJR-GARCH-SGT (1.69515), GARCH-N-NN (1.71390), GARCH-T-NN (1.71509), GARCH-ST-NN (1.71786), GARCH-SGT-NN (1.71812), GJR-GARCH-N-NN (1.68169), GJR-GARCH-T-NN (1.69363), GJR-GARCH-ST-NN (1.69340) and GJR-GARCH-SGT-NN (1.69350). In other words, regarding the TSX, the GJR-GARCH-N model has the lowest value of MAE among all sixteen models. In Table 3 the result ‘2’ is then recorded in column ‘S4’, corresponding to the row ‘GJR-GARCH-N’. In Table 7, the above result ‘2’ is also recorded in column ‘MAE’ below ‘In-sample’ of S4, corresponding to the row ‘GJR-GARCH-N’.

Table 7 lists the summary results of performance comparison for the in-sample and out-of-sample volatility forecasts based on the MAE and RMSE loss functions. In other words, the numbers in column ‘MAE’ below ‘In-sample’ of S1, S2, S3 and S4 are respectively summarized from those in columns ‘S1’, ‘S2’, ‘S3’ and ‘S4’ of Table 3. On the contrary, the numbers in column ‘RMSE’ below ‘In-sample’ of S1, S2, S3 and S4 are respectively summarized from those in columns ‘S1’, ‘S2’, ‘S3’ and ‘S4’ of Table 4. The numbers in column ‘MAE’ below ‘Out-of-sample’ of S1, S2, S3 and S4 are respectively summarized from those in columns ‘S1’, ‘S2’, ‘S3’ and ‘S4’ of Table 5. Conversely, the numbers in column ‘RMSE’ below ‘Out-of-sample’ of S1, S2, S3 and S4 are respectively summarized from those in columns ‘S1’, ‘S2’, ‘S3’ and ‘S4’ of Table 6. In order to easily explore the four main issues of this study, I performed calculations for the summation of all four numbers in column ‘S1’ for each model, as well as columns ‘S2’, ‘S3’ and ‘S4’. For example, regarding the GARCH-N model, the numbers ‘4′ and ‘0’ are respectively in columns ‘MAE’ and ‘RMSE’ below ‘In-sample’ of S1. Moreover, the numbers ‘3′ and ‘5′ are respectively in columns ‘MAE’ and ‘RMSE’ below ‘Out-of-sample’ of S1. Hence, in Table 7, the summation of all four numbers in column ‘S1’ is equal to 12, and is recorded in the column ‘Sum’ below ‘S1’ and the row ‘GARCH-N’. Regarding the other 15 models, the summation of all four numbers in columns ‘S1’ must be done with the same inference process. With regard to the 16 models, the summation of all four numbers in columns ‘S2’, ‘S3’ or ‘S4’ must also be evaluated with the same inference process. The above summation results in columns ‘S2’, ‘S3’ or ‘S4’ are recorded in the column ‘Sum’ below ‘S1’, ‘S3’ or ‘S4’, respectively. Subsequently, I used all 16 numbers in column ‘Sum’ below ‘S1’, ‘S2’, ‘S3’, and ‘S4’ of Table 7 to execute the performance comparison of volatility forecasting for four categories of model. As shown by the numbers at column ‘Sum’ below S1 of Table 7, I found that the numbers for all eight composed volatility forecasting models are far greater than those for all eight corresponding parametric volatility forecasting models. For example, the number for the GARCH-N-NN model (44) is far greater than that for the GARCH-N model (12). These results indicate that the performance for the composed volatility forecasting models is significantly superior to that for the parametric volatility forecasting models. In other words, the neural network approach can significantly improve the performance of volatility forecasting. As reported by the numbers in column ‘Sum’ below S2 of Table 7, I found that, regarding the parametric volatility forecasting approach, the numbers for all four GJR-based models are far greater than those for all four corresponding GARCH-based models, as shown in panel A of this table. For instance, the number for the GJR-GARCH-N model (38) is far greater than that for the GARCH-N model (18). I also found that, regarding the composed volatility forecasting approach, the numbers for all four GJR-based models are far greater than those for all four corresponding GARCH-based models, as shown in panel B of this table. These results imply that irrespective of the parametric forecasting approach or composed forecasting approach, the performance for the GJR-based models is significantly superior to that of the GARCH-based models. That is to say, the setting of the leverage effect can significantly encourage the performance of volatility forecasting (as shown in Section 2, the GJR-based model can seize the leverage effect appearing at the financial assets whereas the GARCH-based model cannot.) As illustrated by the numbers in column ‘Sum’ below S3 of Table 7, I found that the numbers for the models with non-normal distribution are not greater than those for the models with normal distribution based on the same volatility forecasting approach and volatility specification. For example, the number for the GARCH-N model (35) is far greater than those for the GARCH-T (1), GARCH-ST (5) and GARCH-SGT (15) models. Moreover, the number for the GJR-GARCH-N model (45) is far greater than those for the GJR-GARCH-T (2), GJR-GARCH-ST (3) and GJR-GARCH-SGT (6) models. Furthermore, the number for the GARCH-N-NN model (37) is far greater than those for the GARCH-T-NN (13), GARCH-ST-NN (4) and GARCH-SGT-NN (2) models. In addition, the number for the GJR-GARCH-N-NN model (32) is far greater than those for the GJR-GARCH-T-NN (12), GJR-GARCH-ST-NN (3) and GJR-GARCH-SGT-NN (10) models. The above results indicate that irrespective of volatility forecasting approach or volatility specification, the performance of the models with the non-normal distribution is not superior to that of the models with the normal distribution. In other words, the setting of the non-normal return distribution cannot promote the performance of volatility forecasting. As listed by the 16 numbers in column ‘Sum’ below S4 of Table 7, I found the number for the GJR-GARCH-N-NN model (15) is the greatest. On the contrary, the numbers for the GJR-GARCH-N, GJR-GARCH-T-NN and GJR-GARCH-SGT-NN are all equal to 9, the second greatest among the 16 numbers. The above result indicates that, among the 16 models in this study, the performance of the GJR-GARCH-N-NN models is the best followed by GJR-GARCH-N, GJR-GARCH-T-NN and GJR-GARCH-SGT-NN. In other words, the GJR-GARCH model combined with both the normal distribution and a neural networks approach has the best performance of volatility forecasting among the sixteen models in this study.

In addition, this study also investigates which model is the most suitable for each of the fourteen stock indices. That is, regarding each stock index, which model has the best performance of volatility forecasting in order to find the most suitable model for each stock index. In order to easily explore this issue, I summarized the most superior model for each stock index based on two types of volatility forecasts (in-sample and out-of-sample) and two types of loss function (MAE and RMSE). Taking an example of ‘DJ’ stock index, among the 16 models, the GJR-GARCH-SGT-NN model has the best performance for in-sample volatility forecast based on MAE (respectively, RMSE) as shown in the column ‘DJ’ of Table 3 (respectively, Table 4). These results are recorded in column ‘DJ’ and rows ‘MAE’ and ‘RMSE’ of ‘In-sample’ in Table 8. Conversely, among the 16 models, the GJR-GARCH-N-NN model has the best performance for out-of-sample volatility forecast based on MAE (respectively, RMSE) as shown in the column ‘DJ’ of Table 5 (respectively, Table 6). These results are recorded in column ‘DJ’ and rows ‘MAE’ and ‘RMSE’ of ‘Out-of-sample’ in Table 8. Hence, Table 8 summarizes the results of the most suitable mode for alternative stock indices. In other words, the results listed in row ‘MAE’ (respectively, ‘RMSE’) of ‘In-Sample’ in Table 8 are summarized from the results of the performance comparison for the fourth category of model in Table 3 (respectively, Table 4). On the other hand, the results listed in row ‘MAE’ (respectively, ‘RMSE’) of ‘Out-of-Sample’ in Table 8 are summarized from the results of the performance comparison for the fourth category of model in Table 5 (respectively, Table 6). From Table 8, I found that both GJR-GARCH-N-NN and GJR-GARCH-SGT-NN are the most suitable models for the DJ stock index because both the GJR-GARCH-N-NN and GJR-GARCH-SGT-NN appear twice among four cases in column ‘DJ’ in Table 8 (the four cases in Table 8 are composed of two types of volatility forecasts (in-sample and out-of-sample) and two types of loss function (MAE and RMSE) when the volatility forecasting of a specific stock index is executed). These results are recorded in row ‘Best model’, corresponding to column ‘DJ’ in Table 8. On the contrary, GJR-GARCH-N is the most suitable model for the TSX stock index because GJR-GARCH-N is the most relevant for the four cases. These results are recorded in row ‘Best model’, corresponding to column ‘TSX’ in Table 8. In the same inference process, I found the most suitable models for the others stock indices, and I recorded them in row ‘Best model’ and the column corresponding to the specific stock index in Table 8. From the results listed in the row ‘Best model’ of Table 8, I obtained the following conclusion. First, GJR-GARCH-N is the most suitable model for the TSX, BVSP and MXX. Second, GARCH-N-NN is the most suitable model only for the RTSI. Third, GJR-GARCH-N-NN is the most suitable model for the DJ, FTSE, MIB and SSE. Fourth, GJR-GARCH-T-NN is the most suitable model for the DAX, JKSE and XU100. Fifth, the GJR-GARCH-SGT-NN is the most suitable model for the DJ, N225 and BSE. To sum up, the most suitable models for the 14 stock indices are distributed at the GJR-GARCH-N, GARCH-N-NN, GJR-GARCH-N-NN, GJR-GARCH-T-NN and GJR-GARCH-SGT-NN models. These results indicate that the most suitable models are not necessarily the same for each of the 14 stock indices. Regarding the most suitable models above, they possess the setting of leverage effect and further combine with a neural networks approach. As to the setting of distribution, they are randomly distributed at the normal, Student’s t and SGT. Hence, the above conclusions are the same as those obtained from the analysis of previous issues. That is, a neural network approach and the setting of leverage effect can significantly promote the performance of volatility forecasting but the setting of non-normal distribution cannot.

## 5. Conclusions

This study uses the stock indices in the developed and emerging markets as the sample and then utilizes eight parametric volatility forecasting models and eight composed volatility forecasting models to explore whether the neural networks approach can promote the performance of volatility forecasting, whether the settings of leverage effect and non-normal return distribution can encourage the performance of volatility forecasting and which one of the 16 models posseses the best volatility forecasting performance. In addition, this study also investigates which model is the most suitable for each of 14 stock indices. The stock indices in the G7 and E7 are used to represent the stock markets in the developed and emerging markets, respectively. The eight parametric volatility forecasts models are composed of the GJR-GARCH or GARCH models with the normal, Student’s t, skewed Student’s t and generalized skewed Student’s t distributions. The eight composed volatility forecasting models are the eight parametric volatility forecasting models combined with a neural network approach. Notably, the neural network model has the same concepts as the network topology.

The empirical findings can be summarized as follows. From the descriptive statistics of data and estimation results of the GJR-GARCH-SGT model, I obtained the following conclusions. First, the stock indices in the E7 have higher return and higher risk than those in the G7. Second, all the stock indices in the G7 and E7 for the forecasting period have higher risk than those for the overall period because of COVID-19 spreading throughout the world in the last year. Third, the leverage effect exists significantly in the stock indices in the G7 and E7, especially for the G7. Fourth, the distribution of returns is left-skewed and has a larger and thicker tail than the normal distribution. From the performance comparison for the four categories of model, I drew the following conclusions, irrespective of in-sample or out-of-sample volatility forecasting. First, the performance for the composed volatility forecasting models is significantly superior to that of the parametric volatility forecasting models, indicating that the neural network approach can significantly improve the performance of volatility forecasting. Second, irrespective of parametric forecasting approach or composed forecasting approach, the performance for the GJR-based models is significantly superior to that of the GARCH-based models, implying that the setting of the leverage effect can significantly encourage the performance of volatility forecasting. Third, irrespective of volatility forecasting approach or volatility specification, the performance of the models with non-normal distribution are not superior to that of the models with the normal distribution, indicating that the setting of the non-normal return distribution cannot promote the performance of volatility forecasting. Fourth, among the 16 models in this study, the performance of the GJR-GARCH-N-NN models is the best followed by GJR-GARCH-N, GJR-GARCH-T-NN and GJR-GARCH-SGT-NN, inferring that the GJR-GARCH model combined with both the normal distribution and a neural network approach has the better performance of volatility forecasting. Finally, for each of the 14 stock indices, the most suitable models are not necessarily the same but they possess the setting of leverage effect and further combine with a neural network approach, and thus these results are the same a those obtained from the analysis of previous issues.

Based on the above empirical results, I propose the following important policy implications for investors and fund managers. First, the investors should use the asymmetric GARCH model to precisely forecast the volatility of stock indices because the asymmetric GARCH model such as the GJR-based model can seize the leverage effect existing in the stock indices and the setting of the leverage effect can significantly encourage the performance of volatility forecasting. Second, the investors should utilize the composed volatility forecasting models to accurately forecast the volatility of stock indices because the neural networks approach can handle the complex, non-linear univariate and multivariate relationships that would be difficult to fit using other techniques, and therefore it can significantly promote the performance of volatility forecasting. Hence, the two policy implications above can enable investors and fund managers to precisely execute subsequent investment processes such as the asset allocation, option pricing, risk management and hedge strategy in the stock market.

## Figures and Tables

**Table 1:** Descriptive statistics of daily return for the G7 and E7 stock indices.

	Mean	SD	Max.	Min.	SK	KUR	J-B	$Q^{2}\left(20\right)$	Obs.
Panel A. Overall period									
DJ	**0.0263**	1.3563	10.764	−12.577	−0.6470 ^c^	11.298 ^c^	18,866.79 ^c^	4031.63 ^c^	3501
TSX	0.0181	1.2631	11.294	−16.998	−1.3781 ^c^	24.159 ^c^	86,251.62 ^c^	2302.80 ^c^	3501
FTSE	7 × 10^−5^	1.3977	10.870	−11.512	−0.3820 ^c^	8.844 ^c^	11,495.47 ^c^	2956.18 ^c^	3501
CAC40	−0.0039	1.6809	11.285	−13.098	−0.3211 ^c^	6.579 ^c^	6374.96 ^c^	2364.63 ^c^	3501
DAX	0.0198	1.7382	11.588	−13.054	−0.3698 ^c^	6.076 ^c^	5466.49 ^c^	2320.15 ^c^	3501
MIB	−0.0215	**1.8306**	13.761	−18.541	−0.8287 ^c^	10.120 ^c^	15,341.27 ^c^	1130.00 ^c^	3501
N225	0.0146	1.7015	11.805	−12.715	−0.3951 ^c^	6.136 ^c^	5584.40 ^c^	1438.09 ^c^	3501
BVSP	0.0519	2.0885	13.022	−18.749	−0.5818 ^c^	7.231 ^c^	7826.8 ^c^	2207.54 ^c^	3501
RTSI	0.0665	**2.3155**	15.642	−20.779	−0.5733 ^c^	8.425 ^c^	10,546.1 ^c^	803.26 ^c^	3501
BSE	0.0633	1.7259	14.412	−17.183	−0.5704 ^c^	11.296 ^c^	18,804.7 ^c^	777.52 ^c^	3501
SSE	0.0254	1.9996	45.160	−12.763	3.1136 ^c^	77.546 ^c^	882,866.9 ^c^	8.47	3501
MXX	0.0529	1.4361	10.153	−16.277	−0.5429 ^c^	10.026 ^c^	14,837.2 ^c^	1372.47 ^c^	3501
JKSE	**0.0717**	1.6150	13.624	−12.925	−0.3663 ^c^	11.109 ^c^	18,081.8 ^c^	922.40 ^c^	3501
XU100	0.0685	2.2764	15.642	−20.330	−0.4234 ^c^	8.447 ^c^	10,514.0 ^c^	582.22 ^c^	3501
Panel B. Estimation period									
DJ	**0.0280**	1.2554	7.877	−11.269	−0.5146 ^c^	6.8384 ^c^	6478.18 ^c^	2837.12 ^c^	3251
TSX	0.0199	1.1800	8.709	−16.998	−1.1476 ^c^	20.079 ^c^	55,328.6 ^c^	1291.52 ^c^	3251
FTSE	0.0062	1.3506	10.870	−10.327	−0.1799 ^c^	7.4255 ^c^	7486.55 ^c^	3469.63 ^c^	3251
CAC40	−0.0010	1.6441	11.285	−11.476	−0.1231 ^c^	5.4008 ^c^	3959.36 ^c^	2637.30 ^c^	3251
DAX	0.0192	1.7058	11.588	−11.829	−0.2596 ^c^	4.8478 ^c^	3220.02 ^c^	2745.92 ^c^	3251
MIB	−0.0206	**1.7888**	13.761	−13.331	−0.4568 ^c^	6.8566 ^c^	6481.45 ^c^	1414.19 ^c^	3251
N225	0.0144	1.6995	11.805	−12.715	−0.3750 ^c^	6.2068 ^c^	5294.73 ^c^	1379.55 ^c^	3251
BVSP	0.0537	2.0182	10.967	−18.749	−0.4689 ^c^	5.598 ^c^	4365.59 ^c^	1502.78 ^c^	3251
RTSI	0.0678	**2.3383**	15.642	−20.779	−0.5240 ^c^	8.347 ^c^	9587.39 ^c^	805.08 ^c^	3251
BSE	0.0688	1.6886	14.412	−17.183	−0.5087 ^c^	11.009 ^c^	16,558.95 ^c^	632.53 ^c^	3251
SSE	0.0133	1.8765	12.950	−12.763	−0.1915 ^c^	5.443 ^c^	4033.75 ^c^	914.03 ^c^	3251
MXX	0.0627	1.4227	10.153	−16.277	−0.5190 ^c^	10.645 ^c^	15,495.60 ^c^	1239.05 ^c^	3251
JKSE	**0.0842**	1.6079	13.624	−12.925	−0.3487 ^c^	11.231 ^c^	17,152.38 ^c^	810.85 ^c^	3251
XU100	0.0673	2.3067	15.642	−20.330	−0.3862 ^c^	8.431 ^c^	9710.74 ^c^	537.72 ^c^	3251
Panel C. Forecasting period									
DJ	0.0030	2.2948	10.764	−12.577	−0.7590 ^c^	10.203 ^c^	1113.02 ^c^	355.686 ^c^	251
TSX	−0.0036	2.0577	11.294	−13.176	−1.6405 ^c^	18.926 ^c^	3859.00 ^c^	376.123 ^c^	251
FTSE	−0.0767	1.9063	8.666	−11.512	−1.2277 ^c^	10.858 ^c^	1296.15 ^c^	198.535 ^c^	251
CAC40	−0.0401	2.1009	8.056	−13.098	−1.5277 ^c^	11.175 ^c^	1403.71 ^c^	171.667 ^c^	251
DAX	**0.0273**	2.1156	10.414	−13.054	−1.1140 ^c^	12.113 ^c^	1586.48 ^c^	144.527 ^c^	251
MIB	−0.0322	**2.3058**	8.549	−18.541	−3.0502 ^c^	24.071 ^c^	6449.01 ^c^	96.842 ^c^	251
N225	0.0177	1.7269	6.889	−7.142	−0.6490 ^c^	5.439 ^c^	327.02 ^c^	109.667 ^c^	251
BVSP	0.0324	2.8492	13.022	−15.993	−1.0412 ^c^	10.454 ^c^	1188.4 ^c^	343.631 ^c^	251
RTSI	0.0587	1.9986	8.280	−12.049	−1.6007 ^c^	9.016 ^c^	957.3 ^c^	15.876	251
BSE	−0.0077	2.1521	11.573	−11.3274	−0.8935 ^c^	10.961 ^c^	1290.0 ^c^	113.595 ^c^	251
SSE	**0.1812**	**3.1898**	45.160	−6.7124	11.2323 ^c^	159.326 ^c^	270,762 ^c^	0.325	251
MXX	−0.0605	1.6095	5.618	−7.3361	−0.6836 ^c^	4.408 ^c^	222.7 ^c^	206.215 ^c^	251
JKSE	−0.0950	1.6979	9.704	−9.6359	−0.5312 ^c^	10.033 ^c^	1064.5 ^c^	193.267 ^c^	251
XU100	0.0778	1.8411	5.810	−9.6871	−1.3201 ^c^	5.618 ^c^	403.0 ^c^	80.058 ^c^	251

Notes: 1. The superscripts a, b and c denote significance at the 10%, 5% and 1% levels, respectively. 2. SK and KUR denote the skewness and excess kurtosis, respectively. 3. J-B statistics are based on Jarque and Bera (1987) and are asymptotically chi-squared-distributed with 2 degrees of freedom. 4. $Q^{2}\;\left(20\right)$ statistics are asymptotically chi-squared-distributed with 20 degrees of freedom. 5. Obs. denotes the number of observations. 6. The overall period starts from 10 January 2001 to 27 July 2020. On the other hand, the estimation period is from 10 January 2001 to 8 April 2019 whereas the forecasting period is from 9 April 2019 to 27 July 2020. 7. Bold font in column ‘Mean’ (resp. ‘SD’) of each panel denotes the largest value when all seven numbers in column ‘Mean’ (resp. ‘SD’) corresponding to the G7 or E7 are compared with each other. 8. Underline font in column ‘Mean’ (resp. ‘SD’) of each panel denotes the smallest value when all seven numbers in column ‘Mean’ (resp. ‘SD’) corresponding to the G7 or E7 are compared with each other.

**Table 2:** The empirical results of the GJR-GARCH-SGT model for the overall period.

	DJ	TSX	FTSE	CAC40	DAX	MIB	N225
$\phi_{0}$	0.0391 (0.013) ^c^	0.0367 (0.012) ^c^	0.0018 (0.015)	0.0187 (0.018)	0.0377 (0.019) ^a^	0.0177 (0.020)	0.0380 (0.022) ^a^
$\phi_{1}$	−0.0472 (0.016) ^c^	0.0037 (0.016)	−0.0306 (0.016) ^a^	−0.0303 (0.016) ^a^	0.0012 (0.016)	−0.0217 (0.016)	−0.0252 (0.016)
ω	0.0290 (0.004) ^c^	0.0227 (0.004) ^c^	0.0309 (0.005) ^c^	0.0398 (0.004) ^c^	0.0387 (0.006) ^c^	0.0326 (0.006) ^c^	0.0861 (0.016) ^c^
α	−0.0218 (0.008) ^c^	0.0241 (0.009) ^b^	−0.0168 (0.007) ^b^	−0.0222 (0.006) ^c^	−0.0162 (0.005) ^c^	−0.0007 (0.008)	0.0232 (0.009) ^b^
β	0.8929 (0.010) ^c^	0.8837 (0.011) ^c^	0.9008 (0.012) ^c^	0.9026 (0.003) ^c^	0.9121 (0.008) ^c^	0.9150 (0.010) ^c^	0.8732 (0.014) ^c^
η	0.2176 (0.021) ^c^	0.1421 (0.020) ^c^	0.1943 (0.022) ^c^	0.2046 (0.010) ^c^	0.1759 (0.017) ^c^	0.1433 (0.015) ^c^	0.1488 (0.023) ^c^
C	0.9799	0.97885	0.98115	0.9827	0.98385	0.98595	0.9708
n	7.0798 (1.423) ^c^	5.1560 (0.701) ^c^	5.7090 (0.905) ^c^	5.9134 (0.917) ^c^	9.5401 (2.326) ^c^	5.7027 (0.886) ^c^	7.1754 (1.542) ^c^
λ	−0.1229 (0.022) ^c^	−0.1548 (0.023) ^c^	−0.1143 (0.023) ^c^	−0.1380 (0.023) ^c^	−0.1090 (0.021) ^c^	−0.1445 (0.023) ^c^	−0.0554 (0.021) ^b^
κ	1.7311 (0.126) ^c^	2.1707 (0.162) ^c^	2.1041 (0.163) ^c^	2.1338 (0.159) ^c^	1.6614 (0.119) ^c^	2.0680 (0.157) ^c^	1.7058 (0.139) ^c^
*Q^2^* (20)	3.380	39.941 ^c^	12.510	10.813	9.605	48.903 ^c^	7.029
LL	−4974.32	−4631.04	−5207.87	−5922.27	−6058.64	−6215.33	−6306.45
	**BVSP**	**RTSI**	**BSE**	**SSE**	**MXX**	**JKSE**	**XU100**
$\phi_{0}$	0.0565 (0.026) ^b^	0.0991 (0.026) ^c^	0.0778 (0.017) ^c^	0.0116 (0.018)	0.0426 (0.018) ^b^	0.0850 (0.016) ^c^	0.0958 (0.030) ^c^
$\phi_{1}$	−0.0144 (0.016)	0.0102 (0.015)	0.0463 (0.016) ^c^	0.0142 (0.011)	0.0460 (0.016) ^c^	−0.0024 (0.015)	−0.0004 (0.016)
ω	0.0946 (0.020) ^c^	0.1205 (0.011) ^c^	0.0671 (0.012) ^c^	0.0351 (0.011)^c^	0.0360 (0.005) ^c^	0.0613 (0.015) ^c^	0.0960 (0.012) ^c^
α	0.0143 (0.007) ^a^	0.0449 (0.004) ^c^	0.0363 (0.009) ^c^	0.0460 (0.009)^c^	0.0296 (0.008) ^c^	0.0698 (0.013) ^c^	0.0425 (0.006) ^c^
β	0.9197 (0.010) ^c^	0.9002 (0.002) ^c^	0.8701 (0.013) ^c^	0.9235 (0.012)^c^	0.9089 (0.003) ^c^	0.8706 (0.016) ^c^	0.9161 (0.003) ^c^
η	0.0832 (0.015) ^c^	0.0672 (0.007) ^c^	0.1534 (0.026) ^c^	0.0520 (0.016)^c^	0.0873 (0.012) ^c^	0.0824 (0.021) ^c^	0.0455 (0.013) ^c^
C	0.9756	0.9787	0.9831	0.9955	0.98215	0.9816	0.98135
n	4.9911 (0.709) ^c^	4.2549 (0.547) ^c^	3.8139 (0.386) ^c^	6.9225 (1.712) ^c^	4.8618 (0.651) ^c^	4.5756 (0.669) ^c^	4.1622 (0.503) ^c^
λ	−0.0808 (0.022) ^c^	−0.0262 (0.020)	−0.0428 (0.020) ^b^	−0.0070 (0.007)	−0.0784 (0.023) ^c^	−0.0603 (0.018) ^c^	−0.0593 (0.022) ^c^
κ	2.1926 (0.180) ^c^	1.9903 (0.165) ^c^	2.4594 (0.220) ^c^	1.3106 (0.104) ^c^	2.0735 (0.158) ^c^	1.7512 (0.146) ^c^	2.1166 (0.169) ^c^
*Q^2^* (20)	27.438	4.758	2.465	31.257 ^a^	21.581	13.741	6.592
LL	−7048.77	−7280.81	−6022.33	−6451.92	−5582.73	−5838.77	−7254.09

Note: 1. The superscripts a, b and c denote significance at the 10%, 5% and 1% levels, respectively. 2. Numbers in parentheses at the rows of parameters are standard errors. 3. LL indicates the log-likelihood value. 4. *Q*^2^ (20) statistics are asymptotically chi-squared-distributed with 20 degrees of freedom. 5. The numbers in row ‘C’ denote the value of the expression ‘β+α+0.5η‘ and are used to check the constraint ‘β+α+0.5η<1’, the necessary and sufficient condition for the existence of the second moment condition for all GJR-based models. Please see Example 2.1 of Ling and McAleer [26] for more details.

**Table 3:** The results of in-sample volatility forecasts based on the MAE loss function for the overall period.

	DJ	TSX	FTSE	CAC40	DAX	MIB	N225	BVSP	RTSI	BSE	SSE	MXX	JKSE	XU100	S1	S2	S3	S4
Panel A. Parametric volatility forecasting model																		
G-n	**1.94301**	**1.67592**	**2.10058**	3.02461	3.22976	*3.79126*	3.20712	**4.61172**	*6.04452*	*3.36402*	5.50737	2.24133	3.12675	*5.81786*	4	4	7	0
G-t	1.99808	1.72006	2.11773	3.06766	3.26971	*3.73640*	3.23995	**4.65463**	*6.08147*	*3.36680*	5.35293	2.24215	3.17611	*5.81197*	1	4	0	0
G-st	1.98032	**1.70677**	**2.11096**	3.04609	3.24980	*3.70646*	*3.22753*	**4.64909**	*6.07069*	*3.36179*	5.35735	2.24080	3.17068	*5.79920*	3	5	3	0
G-sgt	1.95943	**1.70920**	**2.10996**	3.04463	3.23786	*3.70150*	*3.20270*	**4.66265**	*6.06473*	*3.38882*	5.07430	2.24376	3.11594	*5.82081*	3	5	4	0
GJR-n	*1.90932*	* **1.64781** *	*2.04673*	*2.97039*	*3.19964*	3.79163	*3.18855*	* **4.56913** *	6.10394	3.4345	*5.35130*	*2.21215*	*3.09989*	5.82530	2	**10**	**10**	2
GJR-t	*1.93222*	*1.69383*	*2.06506*	*3.01771*	*3.23644*	3.75008	*3.23776*	*4.61684*	6.15222	3.4073	*5.21295*	*2.22328*	*3.14403*	5.82462	0	**10**	0	0
GJR-st	*1.92962*	***1.68906***	*2.06817*	*3.01458*	*3.23019*	3.73637	3.22787	*4.61636*	6.14280	3.4061	*5.21859*	*2.22640*	*3.14468*	5.81643	1	**9**	3	0
GJR-sgt	*1.91756*	*1.69515*	*2.07205*	*3.01955*	*3.22419*	3.74066	3.20802	*4.63335*	6.14009	3.4585	*4.94037*	*2.23054*	*3.10001*	5.84387	0	**9**	1	0
Panel B. Composed volatility forecasting model																		
G-n-NN	1.94312	1.71390	2.11361	**3.01633**	**3.21802**	**3.64754**	3.17210	4.65849	**6.01401**	**3.33401**	**4.87450**	**2.23829**	**3.03618**	**5.78184**	**10**	0	5	0
G-t-NN	**1.94726**	**1.71509**	**2.11215**	**3.01060**	**3.21702**	**3.65168**	**3.17156**	4.66779	* **6.00547** *	**3.33830**	***4.83083***	**2.23706**	**3.02826**	**5.77879**	**13**	2	**8**	1
G-st-NN	**1.94974**	1.71786	2.11302	**3.01324**	**3.21994**	**3.65578**	**3.17275**	4.67251	***6.00691***	**3.33898**	* **4.83020** *	**2.23795**	**3.02936**	**5.77980**	**11**	2	1	0
G-sgt-NN	**1.94958**	1.71812	2.11348	**3.01321**	**3.21988**	**3.65567**	**3.17288**	4.67957	***6.00691***	**3.33889**	***4.83381***	**2.23791**	**3.02896**	**5.77989**	**11**	2	0	0
GJR-n-NN	***1.88192***	*1.68169*	* **2.03321** *	***2.93390***	***3.14309***	* **3.59397** *	***3.14176***	*4.59718*	***6.01255***	***3.3246***	* **4.82876** *	***2.20981***	***3.01293***	***5.77303***	**12**	**14**	5	3
GJR-t-NN	***1.85525***	***1.69363***	***2.03463***	***2.93273***	* **3.14297** *	***3.59830***	***3.11303***	***4.60219***	**6.00800**	* **3.3004** *	**4.86149**	* **2.20971** *	* **3.00418** *	* **5.76911** *	**14**	**12**	**6**	**5**
GJR-st-NN	***1.85510***	*1.69349*	***2.03404***	* **2.93265** *	***3.14357***	***3.60006***	***3.11200***	***4.60396***	**6.00837**	***3.3007***	**4.85811**	***2.20980***	***3.00526***	***5.76948***	**13**	**12**	1	1
GJR-sgt-NN	* **1.85489** *	***1.69350***	***2.03400***	***2.93292***	***3.14333***	***3.60015***	* **3.11165** *	***4.60426***	**6.00833**	* **3.3004** *	**4.85552**	***2.20983***	***3.00551***	***5.76948***	**14**	**12**	3	3

Note: 1. G-n, G-t, G-st, G-sgt, GJR-n, GJR-t, GJR-st and GJR-sgt respectively denote the GARCH-N, GARCH-T, GARCH-ST, GARCH-SGT, GJR-GARCH-N, GJR-GARCH-T, GJR-GARCH-ST and GJR-GARCH-SGT models. On the other hand, G-n-NN, G-t-NN, G-st-NN, G-sgt-NN, GJR-n-NN, GJR-t-NN, GJR-st-NN and GJR-sgt-NN respectively denote the GARCH-N, GARCH-T, GARCH-ST, GARCH-SGT, GJR-GARCH-N, GJR-GARCH-T, GJR-GARCH-ST and GJR-GARCH-SGT models with a neural network approach. 2. Bold font denotes the lower value of loss function when the predictive accuracies of one parametric volatility forecasting model and one corresponding composed volatility forecasting model are compared with each other based on the same volatility specification and return distribution. 3. Italic font denotes the lower value of loss function when the predictive accuracies of one GARCH-based model and one corresponding GJR-based model are compared with each other based on the same volatility forecasting approach and return distribution. 4. Underline font denotes the lowest value of loss function when the predictive accuracies of a group of models are compared with each other based on the same volatility specification and volatility forecasting approach but the different distribution. 5. Shade font denotes the lowest value of loss function when the predictive accuracies of all sixteen models are compared with each other. 6. The number in column S1 denotes the total number of indices that a specific model possesses, the lower value of loss function when the predictive accuracies of two models are compared with each other as shown by note 2. This result can illustrate the performance comparison of two volatility forecasting models with different volatility forecasting approach, a parametric or composed volatility forecasting approach. 7. The number in column S2 denotes the total number of indices that a specific model possesses, the lower value of loss function when the predictive accuracies of two models are compared with each other as shown by note 3. This result can illustrate the performance comparison of two volatility forecasting models with different volatility specifications, the GJR-GARCH or GARCH. 8. The number in column S3 denotes the total number of indices that a specific model possesses, the lowest value of loss function when the predictive accuracies of four models are compared with each other as shown by note 4. This result can illustrate the performance comparison of a group of volatility forecasting models with different return distributions, the normal, Student’s t, ST, and SGT. 9. The number in column S4 denotes the total number of indices that a specific model possesses, the lowest value of loss function when the predictive accuracies of all sixteen models are compared with each other as shown by note 5.

**Table 4:** The results of in-sample volatility forecasts based on the RMSE loss function for the overall period.

	DJ	TSX	FTSE	CAC40	DAX	MIB	N225	BVSP	RTSI	BSE	SSE	MXX	JKSE	XU100	S1	S2	S3	S4
Panel A. Parametric volatility forecasting model																		
G-n	5.88987	7.46073	5.89514	7.65691	7.96846	*11.2187*	7.78205	12.2476	*16.6405*	*10.4222*	36.8334	6.78058	9.07556	16.2150	0	3	**10**	0
G-t	5.92925	7.49119	5.90315	7.67566	7.97518	*11.1961*	7.79092	12.2757	*16.6594*	*10.4458*	36.1581	6.78075	9.07132	*16.2114*	0	4	0	0
G-st	5.92579	7.49069	5.90379	7.67298	7.97296	*11.1889*	7.79059	12.2870	*16.6587*	*10.4451*	36.1602	6.78240	9.07138	*16.2113*	0	4	1	0
G-sgt	5.91719	7.49141	5.90355	7.67257	7.97120	*11.1871*	7.78675	12.2863	*16.6581*	*10.4502*	36.0788	6.78269	9.05736	*16.2130*	0	4	3	0
GJR-n	*5.70424*	*7.38230*	*5.77953*	*7.57192*	*7.86956*	11.2521	*7.68501*	*12.0622*	16.6892	10.4336	*36.2758*	*6.74763*	*8.98016*	*16.2136*	0	**11**	**11**	0
GJR-t	*5.74383*	*7.43802*	*5.80208*	*7.61511*	*7.88250*	11.2101	*7.70976*	*12.0925*	16.7084	10.4899	*35.8114*	*6.75102*	*8.96937*	16.2183	0	**10**	1	0
GJR-st	*5.74790*	*7.43847*	*5.80758*	*7.62307*	*7.88265*	11.2170	*7.70583*	*12.1009*	16.7058	10.4907	*35.8165*	*6.75344*	*8.97349*	16.2186	0	**10**	0	0
GJR-sgt	*5.73878*	*7.44376*	*5.80979*	*7.62584*	*7.88251*	11.2195	*7.69860*	*12.1022*	16.7053	10.5198	*35.7892*	*6.75423*	*8.96183*	16.2223	0	**10**	2	0
Panel B. Composed volatility forecasting model																		
G-n-NN	**5.88973**	**7.44449**	**5.81596**	**7.61656**	**7.92175**	**11.0607**	**7.76945**	**12.2058**	* **16.6279** *	**10.3714**	**35.5475**	**6.77104**	**9.02154**	**16.1876**	**14**	1	**10**	1
G-t-NN	**5.90828**	**7.45553**	**5.82481**	**7.62301**	**7.92468**	**11.0969**	**7.77131**	**12.2550**	**16.6353**	**10.3811**	**35.5333**	**6.77054**	**9.01135**	**16.1838**	**14**	0	3	0
G-st-NN	**5.91183**	**7.46233**	**5.82701**	**7.62766**	**7.92847**	**11.0989**	**7.77327**	**12.2656**	**16.6360**	**10.3821**	**35.5329**	**6.77200**	**9.01313**	**16.1844**	**14**	0	0	0
G-sgt-NN	**5.90998**	**7.46188**	**5.82649**	**7.62748**	**7.92741**	**11.0978**	**7.77287**	**12.2618**	**16.6360**	**10.3818**	**35.5327**	**6.77197**	**9.01232**	**16.1845**	**14**	0	1	0
GJR-n-NN	***5.66838***	* **7.32958** *	***5.73452***	* **7.47130** *	***7.79466***	* **10.9658** *	***7.64847***	* **12.0412** *	**16.6300**	***10.3704***	* **35.4988** *	* **6.73087** *	***8.94364***	***16.1678***	**14**	**13**	**7**	**6**
GJR-t-NN	***5.65690***	***7.34451***	***5.73045***	***7.47338***	* **7.79334** *	***10.9964***	***7.54694***	***12.0920***	***16.6347***	***10.3346***	***35.5085***	***6.73146***	* **8.91664** *	* **16.1633** *	**14**	**14**	3	3
GJR-st-NN	***5.65575***	***7.34291***	***5.72990***	***7.47311***	***7.79390***	***10.9981***	***7.54500***	***12.1007***	***16.6351***	***10.3352***	***35.5083***	***6.73169***	***8.91944***	***16.1634***	**14**	**14**	0	0
GJR-sgt-NN	* **5.65494** *	***7.34184***	* **5.72915** *	***7.47296***	***7.79344***	***10.9985***	* **7.54468** *	***12.1021***	***16.6351***	* **10.3343** *	***35.5044***	***6.73176***	***8.92064***	***16.1634***	**14**	**14**	4	4

Note: 1. G-n, G-t, G-st, G-sgt, GJR-n, GJR-t, GJR-st and GJR-sgt respectively denote the GARCH-N, GARCH-T, GARCH-ST, GARCH-SGT, GJR-GARCH-N, GJR-GARCH-T, GJR-GARCH-ST and GJR-GARCH-SGT models. On the other hand, G-n-NN, G-t-NN, G-st-NN, G-sgt-NN, GJR-n-NN, GJR-t-NN, GJR-st-NN and GJR-sgt-NN respectively denote the GARCH-N, GARCH-T, GARCH-ST, GARCH-SGT, GJR-GARCH-N, GJR-GARCH-T, GJR-GARCH-ST and GJR-GARCH-SGT models with a neural network approach. 2. Bold font denotes the lower value of loss function when the predictive accuracies of one parametric volatility forecasting model and one corresponding composed volatility forecasting model are compared with each other based on the same volatility specification and return distribution. 3. Italic font denotes the lower value of loss function when the predictive accuracies of one GARCH-based model and one corresponding GJR-based model are compared with each other based on the same volatility forecasting approach and return distribution. 4. Underline font denotes the lowest value of loss function when the predictive accuracies of a group of models are compared with each other based on the same volatility specification and volatility forecasting approach but the different distribution. 5. Shade font denotes the lowest value of loss function when the predictive accuracies of all sixteen models are compared with each other. 6. The number in column S1 denotes the total number of indices that a specific model possesses, the lower value of loss function when the predictive accuracies of two models are compared with each other as shown by note 2. This result can illustrate the performance comparison of two volatility forecasting models with different volatility forecasting approach, a parametric or composed volatility forecasting approach. 7. The number in column S2 denotes the total number of indices that a specific model possesses, the lower value of loss function when the predictive accuracies of two models are compared with each other as shown by note 3. This result can illustrate the performance comparison of two volatility forecasting models with different volatility specifications, the GJR-GARCH or GARCH. 8. The number in column S3 denotes the total number of indices that a specific model possesses, the lowest value of loss function when the predictive accuracies of four models are compared with each other as shown by note 4. This result can illustrate the performance comparison of a group of volatility forecasting models with different return distributions, the normal, Student’s t, ST, and SGT. 9. The number in column S4 denotes the total number of indices that a specific model possesses, the lowest value of loss function when the predictive accuracies of all sixteen models are compared with each other as shown by note 5.

**Table 5:** The results of out-of-sample volatility forecasts based on the MAE loss function for the forecasting period.

	DJ	TSX	FTSE	CAC40	DAX	MIB	N225	BVSP	RTSI	BSE	SSE	MXX	JKSE	XU100	S1	S2	S3	S4
Panel A. Parametric volatility forecasting model																		
G-n	**5.23835**	**4.08614**	*4.00723*	*4.88746*	*5.25529*	*6.87391*	3.58023	**8.45408**	*5.21086*	*5.54409*	19.7119	2.53884	*3.21863*	*4.25112*	3	8	8	0
G-t	5.48781	**4.34579**	*4.10754*	*5.05897*	*5.41590*	*6.80322*	3.61923	**8.70583**	*5.20198*	*5.57355*	17.8097	***2.53597***	*3.28406*	***4.28156***	4	**9**	0	0
G-st	5.45056	**4.33698**	*4.09935*	*5.04040*	*5.39462*	*6.77097*	3.60625	**8.74539**	*5.18925*	*5.57756*	17.7633	* **2.53355** *	*3.27678*	***4.27488***	4	**9**	1	0
G-sgt	5.39303	**4.33220**	*4.09407*	*5.02931*	*5.36438*	*6.75901*	3.57434	**8.75180**	*5.18462*	*5.61967*	17.1715	***2.53716***	*3.20656*	*4.28206*	3	**9**	5	0
GJR-n	*4.81345*	* **3.77181** *	4.36983	5.20351	5.44052	7.41404	* **3.53313** *	* **8.14552** *	5.45733	5.66595	*17.8680*	* **2.52363** *	3.22240	4.31663	4	6	**13**	3
GJR-t	*4.97463*	***3.99525***	4.40601	5.38883	5.60222	7.47680	*3.56840*	***8.40549***	5.57311	5.74500	*15.8210*	2.54620	3.28613	**4.37388**	3	5	0	0
GJR-st	*4.97868*	***3.99444***	4.43572	5.42069	5.62017	7.51899	*3.56041*	***8.46082***	5.56537	5.76024	*15.8078*	2.55058	3.28866	**4.37328**	3	5	0	0
GJR-sgt	*4.94163*	***3.99792***	4.44068	5.42710	5.60309	7.52452	***3.54297***	***8.47717***	5.56592	5.85565	*15.4829*	2.55956	3.23212	**4.39085**	4	5	1	0
Panel B. Composed volatility forecasting model																		
G-n-NN	5.24135	4.26528	* **3.92162** *	* **4.68967** *	**4.94144**	* **6.13239** *	***3.54364***	8.78185	* **5.14861** *	* **5.14944** *	***11.6175***	**2.53873**	**3.03769**	* **4.23808** *	**11**	**8**	**12**	**6**
G-t-NN	**5.34439**	4.39402	***4.00536***	***4.72423***	**5.03107**	***6.20466***	**3.53207**	9.00670	***5.18240***	***5.30239***	**11.6262**	2.54594	**3.03722**	*4.30382*	**10**	6	1	0
G-st-NN	**5.35238**	4.43273	***3.99540***	**4.78152**	***5.00909***	***6.23998***	**3.57905**	9.17592	***5.16946***	***5.30356***	* **11.3741** *	2.54548	***3.06566***	*4.29301*	**10**	**8**	1	1
G-sgt-NN	**5.36960**	4.45364	***4.02441***	**4.78124**	**5.04596**	***6.20504***	***3.54173***	9.07422	***5.16616***	***5.30945***	***11.5982***	2.54708	**3.11389**	***4.28028***	**11**	7	0	0
GJR-n-NN	* **4.40175** *	*3.94553*	**4.00938**	**4.72984**	* **4.94126** *	**6.24450**	3.60694	*8.36262*	**5.39025**	**5.23549**	**11.8965**	*2.52882*	* **2.99607** *	**4.29655**	**10**	6	**11**	3
GJR-t-NN	***4.55368***	*4.08222*	**4.04434**	**4.74660**	***5.02959***	**6.39646**	* **3.49599** *	*8.68141*	**5.47566**	**5.33312**	* **11.5189** *	***2.54544***	***3.02102***	4.39763	**11**	**8**	2	1
GJR-st-NN	***4.61519***	*4.07934*	**4.11441**	***4.74246***	**5.04989**	**6.48982**	***3.50090***	*8.73967*	**5.47415**	**5.33983**	**12.5474**	***2.54155***	**3.16545**	4.39122	**11**	6	0	0
GJR-sgt-NN	***4.60464***	*4.07151*	**4.11678**	* **4.70148** *	***5.00459***	**6.58809**	3.59051	*8.81177*	**5.47806**	**5.33477**	**11.6727**	***2.54063***	***3.01503***	4.39734	**10**	7	1	0

Note: 1. G-n, G-t, G-st, G-sgt, GJR-n, GJR-t, GJR-st and GJR-sgt respectively denote the GARCH-N, GARCH-T, GARCH-ST, GARCH-SGT, GJR-GARCH-N, GJR-GARCH-T, GJR-GARCH-ST and GJR-GARCH-SGT models. On the other hand, G-n-NN, G-t-NN, G-st-NN, G-sgt-NN, GJR-n-NN, GJR-t-NN, GJR-st-NN and GJR-sgt-NN respectively denote the GARCH-N, GARCH-T, GARCH-ST, GARCH-SGT, GJR-GARCH-N, GJR-GARCH-T, GJR-GARCH-ST and GJR-GARCH-SGT models with a neural network approach. 2. Bold font denotes the lower value of loss function when the predictive accuracies of one parametric volatility forecasting model and one corresponding composed volatility forecasting model are compared with each other based on the same volatility specification and return distribution. 3. Italic font denotes the lower value of loss function when the predictive accuracies of one GARCH-based model and one corresponding GJR-based model are compared with each other based on the same volatility forecasting approach and return distribution. 4. Underline font denotes the lowest value of loss function when the predictive accuracies of a group of models are compared with each other based on the same volatility specification and volatility forecasting approach but the different distribution. 5. Shade font denotes the lowest value of loss function when the predictive accuracies of all sixteen models are compared with each other. 6. The number in column S1 denotes the total number of indices that a specific model possesses, the lower value of loss function when the predictive accuracies of two models are compared with each other as shown by note 2. This result can illustrate the performance comparison of two volatility forecasting models with different volatility forecasting approach, a parametric or composed volatility forecasting approach. 7. The number in column S2 denotes the total number of indices that a specific model possesses, the lower value of loss function when the predictive accuracies of two models are compared with each other as shown by note 3. This result can illustrate the performance comparison of two volatility forecasting models with different volatility specifications, the GJR-GARCH or GARCH. 8. The number in column S3 denotes the total number of indices that a specific model possesses, the lowest value of loss function when the predictive accuracies of four models are compared with each other as shown by note 4. This result can illustrate the performance comparison of a group of volatility forecasting models with different return distributions, the normal, Student’s t, ST, and SGT. 9. The number in column S4 denotes the total number of indices that a specific model possesses, the lowest value of loss function when the predictive accuracies of all sixteen models are compared with each other as shown by note 5.

**Table 6:** The results of out-of-sample volatility forecasts based on the RMSE loss function for the forecasting period.

	DJ	TSX	FTSE	CAC40	DAX	MIB	N225	BVSP	RTSI	BSE	SSE	MXX	JKSE	XU100	S1	S2	S3	S4
Panel A. Parametric volatility forecasting model																		
G-n	15.7616	**16.4357**	12.2028	*14.9531*	15.8453	*26.2827*	7.65260	**25.2073**	* **13.0475** *	15.5771	133.482	**6.00390**	9.18619	**8.79046**	5	3	**10**	1
G-t	15.8326	**16.5444**	*12.2558*	*15.0491*	15.9001	*26.3276*	7.65756	**25.3208**	***13.0901***	**15.6034**	130.953	**5.99677**	9.17535	**8.84355**	6	4	1	0
G-st	15.8345	**16.5793**	*12.2563*	*15.0556*	15.9054	*26.3198*	**7.65689**	**25.3994**	***13.0874***	15.6054	130.920	**5.99752**	9.17864	**8.84692**	6	4	0	0
G-sgt	**15.8172**	**16.5795**	*12.2550*	*15.0514*	15.8985	*26.3137*	7.65226	**25.3976**	***13.0867***	15.6098	130.712	**5.99771**	9.16410	**8.84833**	6	4	3	0
GJR-n	*14.7082*	* **15.4637** *	*12.1957*	15.1460	*15.6646*	26.7312	* **7.54351** *	* **24.1080** *	13.2198	*15.3434*	*131.852*	* **5.94791** *	***8.99512***	* **8.73118** *	6	**11**	**11**	**4**
GJR-t	*14.8234*	***15.6328***	12.2648	15.4193	*15.7535*	26.7665	*7.58346*	***24.1723***	13.3201	* **15.2647** *	*129.895*	*5.95827*	***8.95443***	***8.77517***	5	**10**	1	1
GJR-st	*14.8218*	***15.6234***	12.2999	15.4737	*15.7698*	26.8319	*7.57574*	***24.2560***	13.3142	***15.2693***	*129.888*	*5.96144*	***8.96129***	***8.77606***	5	**10**	0	0
GJR-sgt	*14.8051*	***15.6236***	12.3048	15.4815	*15.7660*	26.8366	*7.56853*	***24.2522***	13.3144	***15.2992***	*129.857*	*5.96565*	* **8.94753** *	***8.78103***	5	**10**	2	1
Panel B. Composed volatility forecasting model																		
G-n-NN	**15.7158**	16.7954	**11.9357**	**14.8658**	**15.7203**	**25.9199**	**7.64866**	25.2355	*13.0734*	**15.5384**	***128.780***	6.01051	**9.16953**	8.84951	**9**	2	**10**	0
G-t-NN	**15.8096**	17.0290	**12.1767**	**14.8586**	**15.7651**	**26.0282**	**7.65025**	25.7240	*13.0932*	**15.6256**	**128.775**	6.01331	**9.16124**	8.89737	**8**	1	1	0
G-st-NN	**15.8205**	17.0909	**12.0342**	**14.9238**	**15.6385**	**26.0064**	7.66164	25.7570	*13.0947*	**15.5870**	***128.742***	6.01462	**9.15128**	8.90285	**8**	2	2	0
G-sgt-NN	15.8872	17.0906	**12.1863**	**14.9109**	**15.7696**	**26.0238**	**7.65046**	25.7959	*13.0877*	**15.6027**	* 128.736 *	6.01486	**9.15479**	8.90320	**8**	2	1	1
GJR-n-NN	* **14.5880** *	*16.0961*	* **11.6052** *	***14.5095***	***15.3074***	* **25.7060** *	*7.58093*	*24.2990*	**13.1519**	* **15.3102** *	**128.813**	*5.94958*	*9.08563*	*8.76723*	**8**	**12**	**9**	3
GJR-t-NN	***14.7197***	*16.2601*	***11.7272***	***14.5034***	***15.3502***	***25.8360***	***7.52585***	*24.8464*	**13.1947**	*15.3500*	* **128.741** *	***5.95229***	*9.05318*	*8.82244*	**9**	**13**	1	0
GJR-st-NN	***14.6896***	*16.2826*	***11.8397***	***14.4597***	***15.3563***	***25.8105***	* **7.52580** *	*24.9499*	**13.1995**	*15.3505*	**128.930**	***5.94996***	*8.99093*	*8.82167*	**9**	**12**	2	1
GJR-sgt-NN	***14.6791***	*16.2717*	***11.8540***	* **14.2583** *	* **15.1765** *	***25.8009***	***7.55066***	*24.9547*	**13.2014**	*15.3489*	**128.759**	***5.95011***	*9.05288*	*8.82304*	**9**	**12**	2	2

Note: 1. G-n, G-t, G-st, G-sgt, GJR-n, GJR-t, GJR-st and GJR-sgt respectively denote the GARCH-N, GARCH-T, GARCH-ST, GARCH-SGT, GJR-GARCH-N, GJR-GARCH-T, GJR-GARCH-ST and GJR-GARCH-SGT models. On the other hand, G-n-NN, G-t-NN, G-st-NN, G-sgt-NN, GJR-n-NN, GJR-t-NN, GJR-st-NN and GJR-sgt-NN respectively denote the GARCH-N, GARCH-T, GARCH-ST, GARCH-SGT, GJR-GARCH-N, GJR-GARCH-T, GJR-GARCH-ST and GJR-GARCH-SGT models with a neural network approach. 2. Bold font denotes the lower value of loss function when the predictive accuracies of one parametric volatility forecasting model and one corresponding composed volatility forecasting model are compared with each other based on the same volatility specification and return distribution. 3. Italic font denotes the lower value of loss function when the predictive accuracies of one GARCH-based model and one corresponding GJR-based model are compared with each other based on the same volatility forecasting approach and return distribution. 4. Underline font denotes the lowest value of loss function when the predictive accuracies of a group of models are compared with each other based on the same volatility specification and volatility forecasting approach but the different distribution. 5. Shade font denotes the lowest value of loss function when the predictive accuracies of all sixteen models are compared with each other. 6. The number in column S1 denotes the total number of indices that a specific model possesses, the lower value of loss function when the predictive accuracies of two models are compared with each other as shown by note 2. This result can illustrate the performance comparison of two volatility forecasting models with different volatility forecasting approach, a parametric or composed volatility forecasting approach. 7. The number in column S2 denotes the total number of indices that a specific model possesses, the lower value of loss function when the predictive accuracies of two models are compared with each other as shown by note 3. This result can illustrate the performance comparison of two volatility forecasting models with different volatility specifications, the GJR-GARCH or GARCH. 8. The number in column S3 denotes the total number of indices that a specific model possesses, the lowest value of loss function when the predictive accuracies of four models are compared with each other as shown by note 4. This result can illustrate the performance comparison of a group of volatility forecasting models with different return distributions, the normal, Student’s t, ST, and SGT. 9. The number in column S4 denotes the total number of indices that a specific model possesses, the lowest value of loss function when the predictive accuracies of all sixteen models are compared with each other as shown by note 5.

**Table 7:** The summary results of performance comparison for the in-sample and out-of-sample volatility forecasts.

	S1					S2					S3					S4				
	In-Sample		Out-of-Sample		Sum	In-Sample		Out-of-Sample		Sum	In-Sample		Out-of-Sample		Sum	In-Sample		Out-of-Sample		Sum
	MAE	RMSE	MAE	RMSE		MAE	RMSE	MAE	RMSE		MAE	RMSE	MAE	RMSE		MAE	RMSE	MAE	RMSE	
Panel A. Parametric volatility forecasting model																				
G-n	4	0	3	5	12	4	3	8	3	18	**7**	**10**	**8**	**10**	**35**	0	0	0	1	1
G-t	1	0	4	6	11	4	4	**9**	4	21	0	0	0	1	1	0	0	0	0	0
G-st	3	0	4	6	13	5	4	**9**	4	22	3	1	1	0	5	0	0	0	0	0
G-sgt	3	0	3	6	12	5	4	**9**	4	22	4	3	5	3	15	0	0	0	0	0
GJR-n	2	0	4	6	12	**10**	**11**	6	**11**	**38**	**10**	**11**	**13**	**11**	**45**	2	0	3	**4**	9
GJR-t	0	0	3	5	8	**10**	**10**	5	**10**	**35**	0	1	0	1	2	0	0	0	1	1
GJR-st	1	0	3	5	9	**9**	**10**	5	**10**	**34**	3	0	0	0	3	0	0	0	0	0
GJR-sgt	0	0	4	5	9	**9**	**10**	5	**10**	**34**	1	2	1	2	6	0	0	0	1	1
Panel B. Composed volatility forecasting model																				
G-n-NN	**10**	**14**	**11**	**9**	**44**	0	1	**8**	2	11	5	**10**	**12**	**10**	**37**	0	1	**6**	0	7
G-t-NN	**13**	**14**	**10**	**8**	**45**	2	0	6	1	9	**8**	3	1	1	13	1	0	0	0	1
G-st-NN	**11**	**14**	**10**	**8**	**43**	2	0	**8**	2	12	1	0	1	2	4	0	0	1	0	1
G-sgt-NN	**11**	**14**	**11**	**8**	**44**	2	0	7	2	11	0	1	0	1	2	0	0	0	1	1
GJR-n-NN	**12**	**14**	**10**	**8**	**44**	**14**	**13**	6	**12**	**45**	5	**7**	**11**	**9**	**32**	3	**6**	3	3	**15**
GJR-t-NN	**14**	**14**	**11**	**9**	**48**	**12**	**14**	**8**	**13**	**47**	**6**	3	2	1	12	**5**	3	1	0	9
GJR-st-NN	**13**	**14**	**11**	**9**	**47**	**12**	**14**	6	**12**	**44**	1	0	0	2	3	1	0	0	1	2
GJR-sgt-NN	**14**	**14**	**10**	**9**	**47**	**12**	**14**	7	**12**	**45**	3	4	1	2	10	3	4	0	2	9

Note: 1. The numbers in column ‘MAE’ below ‘In-sample’ of S1, S2, S3 and S4, respectively are summarized from those in columns ‘S1’, ‘S2’, ‘S3’ and ‘S4’ of Table 3. 2. The numbers in column ‘RMSE’ below ‘In-sample’ of S1, S2, S3 and S4, respectively are summarized from those in columns ‘S1’, ‘S2’, ‘S3’ and ‘S4’ of Table 4. 3. The numbers in column ‘MAE’ below ‘Out-of-sample’ of S1, S2, S3 and S4, respectively are summarized from those in columns ‘S1’, ‘S2’, ‘S3’ and ‘S4’ of Table 5. 4. The numbers in column ‘RMSE’ below ‘Out-of-sample’ of S1, S2, S3 and S4, respectively are summarized from those in columns ‘S1’, ‘S2’, ‘S3’ and ‘S4’ of Table 6. 5. The numbers in column ‘Sum’ below ‘S1’ denote the summation of four numbers in columns ‘MAE’ and ‘RMSE’ below ‘In-sample’ and ‘Out-of-sample’ of S1. 6. The numbers in column ‘Sum’ below ‘S2’ denote the summation of four numbers in columns ‘MAE’ and ‘RMSE’ below ‘In-sample’ and ‘Out-of-sample’ of S2. 7. The numbers in column ‘Sum’ below ‘S3’ denote the summation of four numbers in columns ‘MAE’ and ‘RMSE’ below ‘In-sample’ and ‘Out-of-sample’ of S3. 8. The numbers in column ‘Sum’ below ‘S4’ denote the summation of four numbers in columns ‘MAE’ and ‘RMSE’ below ‘In-sample’ and ‘Out-of-sample’ of S4. 9. Bold font in column ‘S1’ denotes the larger number when two numbers at the same column are compared with each other where two numbers respectively correspond to a parametric volatility forecasting model and its corresponding composed volatility forecasting model, such as the GARCH-N and GARCH-N-N models. 10. Bold font in column ‘S2’ denotes the larger number when two numbers at the same column are compared with each other where two numbers respectively correspond to a GARCH-based model and its corresponding GJR-based model, such as the GARCH-N and GJR-GARCH-N models. 11. Bold font in column ‘S3’ denotes the largest number when four numbers at the same column are compared with each other where four numbers respectively correspond to four models with the same volatility specification and volatility forecasting approach but different return distributions. 12. Bold font in column ‘S4’ denotes the largest number when all sixteen numbers in the same column are compared with each other where sixteen numbers respectively correspond to sixteen models.

**Table 8:** The summary results of the best volatility forecasting model for alternative stock indices.

		DJ	TSX	FTSE	CAC40	DAX	MIB	N225
In-Sample	MAE	**GJR-sgt-NN**	**GJR-n**	**GJR-n-NN**	GJR-st-NN	**GJR-t-NN**	**GJR-n-NN**	**GJR-sgt-NN**
	RMSE	**GJR-sgt-NN**	GJR-n-NN	GJR-sgt-NN	GJR-n-NN	**GJR-t-NN**	**GJR-n-NN**	**GJR-sgt-NN**
Out-of-Sample	MAE	**GJR-n-NN**	**GJR-n**	G-n-NN	G-n-NN	GJR-n-NN	G-n-NN	GJR-t-NN
	RMSE	**GJR-n-NN**	**GJR-n**	**GJR-n-NN**	GJR-sgt-NN	GJR-sgt-NN	**GJR-n-NN**	GJR-st-NN
Best model		GJR-n-NN; GJR-sgt-NN	GJR-n	GJR-n-NN	?	GJR-t-NN	GJR-n-NN	GJR-sgt-NN
		BVSP	RTSI	BSE	SSE	MXX	JKSE	XU100
In-Sample	MAE	**GJR-n**	G-t-NN	GJR-t-NN; **GJR-sgt-NN**	**GJR-n-NN**	GJR-t-NN	**GJR-t-NN**	**GJR-t-NN**
	RMSE	GJR-n-NN	**G-n-NN**	**GJR-sgt-NN**	**GJR-n-NN**	GJR-n-NN	**GJR-t-NN**	**GJR-t-NN**
Out-of-Sample	MAE	**GJR-n**	**G-n-NN**	G-n-NN	G-st-NN	**GJR-n**	GJR-n-NN	G-n-NN
	RMSE	**GJR-n**	G-n	GJR-t	G-sgt-NN	**GJR-n**	GJR-sgt	GJR-n
Best model		GJR-n	G-n-NN	GJR-sgt-NN	GJR-n-NN	GJR-n	GJR-t-NN	GJR-t-NN

Note: 1. This table summarizes the results of the best volatility forecasting model for alternative stock indices in Table 3, Table 4, Table 5 and Table 6. In other words, the results listed in row ‘MAE’ (respectively, ‘RMSE’) of ‘In-Sample’ in Table 8 are summarized from the results of the performance comparison for the fourth category of model in Table 3 (respectively, Table 4). On the other hand, the results listed in row ‘MAE’ (respectively, ‘RMSE’) of ‘Out-of-Sample’ in Table 8 are summarized from the results of the performance comparison for the fourth category of model in Table 5 (respectively, Table 6). 2. GJR-n represents the GJR-GARCH-N model. On the contrary, G-n-NN, GJR-n-NN, GJR-t-NN and GJR-sgt-NN denote the GARCH-N, GJR-GARCH-N, GJR-GARCH-T and GJR-GARCH-SGT models with a neural networks approach. 3. The models listed in row ‘Best model’ and the column corresponding to a specific stock index denote the suitable models for this specific stock index. 4. The symbol ‘?’ in the row ‘Best model’ denotes I could not obtain a suitable model because there is no model that can simultaneously appear twice or more. 5. The bold font in rows ‘MAE’ and ‘RMSE’ of ‘In-sample’ (or ‘Out-of-sample’) denote the suitable models for a specific stock index because for the above four cases this model can simultaneously appear twice or more.

## Data Availability

All the daily close price data of 14 stock indices was obtained from the Yahoo finance website. http://finance.yahoo.com (accessed on 28 July 2021).
